# Measured Canadian oil sands CO_2_ emissions are higher than estimates made using internationally recommended methods

**DOI:** 10.1038/s41467-019-09714-9

**Published:** 2019-04-23

**Authors:** John Liggio, Shao-Meng Li, Ralf M. Staebler, Katherine Hayden, Andrea Darlington, Richard L. Mittermeier, Jason O’Brien, Robert McLaren, Mengistu Wolde, Doug Worthy, Felix Vogel

**Affiliations:** 1 Air Quality Research Division, Environment and Climate Change Canada, 4905 Dufferin Street, Toronto, ON M3H 5T4 Canada; 2 Centre for Atmospheric Chemistry, York University, 4700 Keele Street, Toronto, ON M3J 1P3 Canada; 3 Flight Research Laboratory, National Research Council Canada, Ottawa, ON K1A 0R6 Canada; 4 Climate Research Division, Environment and Climate Change Canada, 4905 Dufferin Street, Toronto, ON M3H 5T4 Canada

**Keywords:** Attribution, Climate-change mitigation

## Abstract

The oil and gas (O&G) sector represents a large source of greenhouse gas (GHG) emissions globally. However, estimates of O&G emissions rely upon bottom-up approaches, and are rarely evaluated through atmospheric measurements. Here, we use aircraft measurements over the Canadian oil sands (OS) to derive the first top-down, measurement-based determination of the their annual CO_2_ emissions and intensities. The results indicate that CO_2_ emission intensities for OS facilities are 13–123% larger than those estimated using publically available data. This leads to 64% higher annual GHG emissions from surface mining operations, and 30% higher overall OS GHG emissions (17 Mt) compared to that reported by industry, despite emissions reporting which uses the most up to date and recommended bottom-up approaches. Given the similarity in bottom-up reporting methods across the entire O&G sector, these results suggest that O&G CO_2_ emissions inventory data may be more uncertain than previously considered.

## Abstract (web summary)

Evaluating GHG emissions reported to inventories for the oil and gas (O&G) sector is important for countries with resource-based economies. Here the authors provide a top-down assessment of GHG emissions from the Canadian oil sands and find previous inventory reports underestimate emissions, by as much as 64% for surface mining facilities and 30% for the entire oil sands compared with their assessment.

## Introduction

The objective of limiting the increase in global temperature to <1.5 °C this century is dependent upon reducing anthropogenic greenhouse gas (GHG) emissions to net zero^1^. Identifying mitigation potentials to achieve this goal and assessing GHG reductions relies upon accurate emission estimates of anthropogenic sources, which inform national and international climate policies. Consequently, participating countries in the United Nations Framework Convention on Climate Change (UNFCCC) are required to submit sector-based emissions data in an annual national GHG inventory report (NIR)^2,3^. Such anthropogenic GHG emission data ultimately underpin carbon pricing and trading policies.

The global energy industry alone accounts for ≈35% of anthropogenic GHG emissions (using a 100-year global warming potential (GWP))^4^, a significant portion of which are attributed to the global upstream oil and gas (O&G) sector. It is estimated that the global O&G sector (which is included in NIRs) contributes ~10% of anthropogenic GHG emissions^4^, using GHG emission estimates that are derived primarily through complex calculations based upon UNFCCC inventory guidelines^5^. The estimation of anthropogenic GHG emissions in NIRs from stationary sources in the energy sector (including the O&G sector) is primarily a bottom–up exercise, where GHG emissions from individual sources within a given facility are added together, as outlined in UNFCCC protocols^5^. These estimates have varying degrees of accuracy (i.e., Tier 1–3)^5^ but fundamentally rely on emission factors and associated activity data (e.g., production and consumption intensity), which in some cases may be outdated or assumed to be homogeneous across the world. In general, Tier 3 approaches are considered to use the best available data specific to the industry and should provide improved emissions estimates relative to Tier 1 and Tier 2 methods.

The large contribution of the O&G sector to global GHG emissions underscores the need for accurate sectoral GHG emissions in national inventories. This is particularly relevant since studies have indicated discrepancies between top–down and bottom–up estimates of O&G methane (CH_4_)^6–12^, many of which having shown that official inventories or industrial reports underestimate emissions. However, few atmospheric measurement-based evaluations have been performed for O&G carbon dioxide (CO_2_) emission inventories, despite CO_2_ emissions being significantly larger than CH_4_. This may be in part because uncertainties in emission factors and activity data used in bottom–up CO_2_ estimates for this sector are considered small (<10%)^5^ compared to CH_4_ (50–150%)^5^. Nonetheless, small uncertainties in CO_2_ emission estimates have the potential to result in large unaccounted for CO_2_ based upon the magnitude of the CO_2_ emitted compared to CH_4_.

Evaluating GHG emissions reported to inventories for the O&G sector is especially important now, with global fossil fuel production and demand at an all-time high^13^. Such evaluations are highly important for countries with resource-based economies such as Canada, where O&G activities account for ≈26% of national GHG emissions and oil sands (OS) production in particular ≈10%^14^. In general, CO_2_ emissions from the O&G sector (including the Canadian OS sector) are derived for inventories using an Intergovernmental Panel on Climate Change (IPCC) Tier 3 approach^15^ and following the UNFCCC protocols^16^. However, estimates of the emission intensity (kg CO_2_e per barrel of oil) of OS operations and of their absolute GHG emissions are not derived from direct measurements of CO_2_. Aircraft measurements over large-scale O&G emission sources can provide a top–down assessment of these bottom–up reported GHG estimates.

Here the utility of a top–down measurement approach is demonstrated below for the Canadian OS surface mining operations (one of the largest single O&G sources of GHGs in Canada). The results indicate that overall, OS GHG emissions may be underestimated and suggests that reporting that follows this Tier 3 approach (or less accurate Tier 1 and Tier 2 approaches) may universally underestimate CO_2_ emissions. This highlights the potential need for updated IPCC inventory guidelines that include atmospheric measurement-based evaluations of CO_2_ emissions, for an improved assessment of the O&G contribution to the global anthropogenic GHG burden.

## Results

### Hourly emission rates

**Fig. 1:**
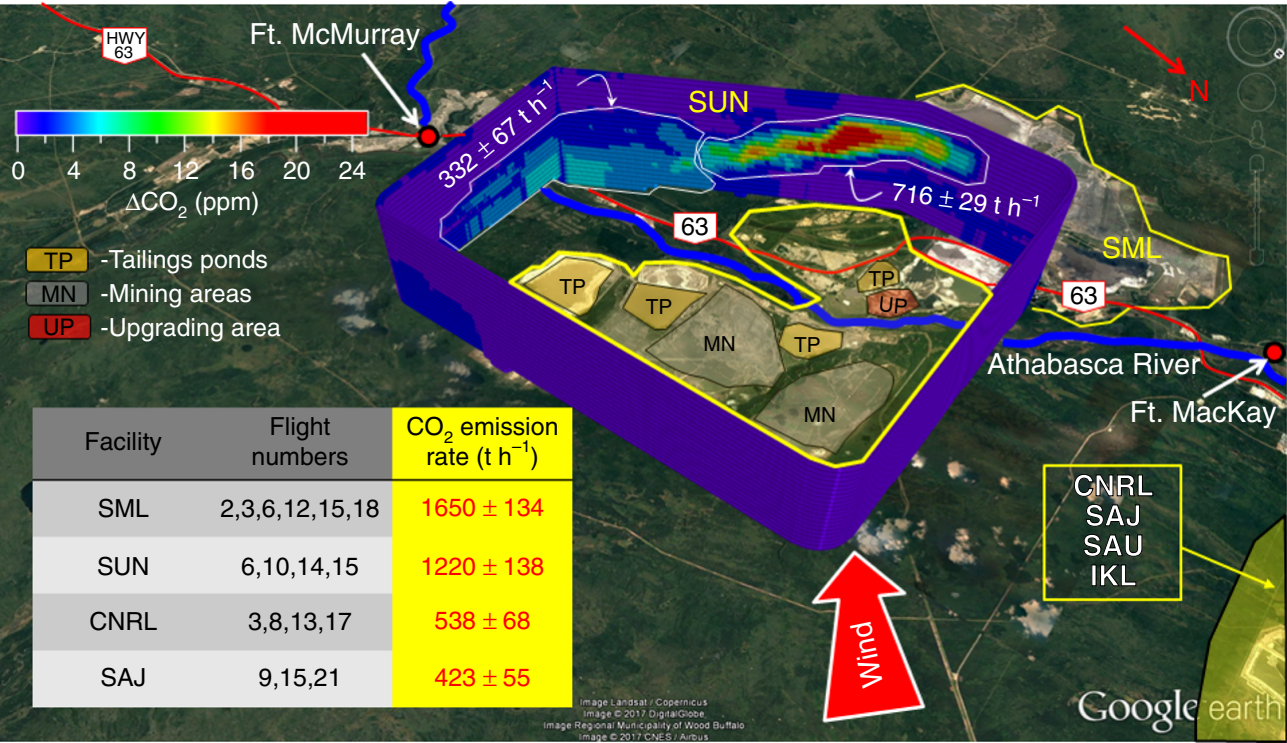
Measured CO_2_ from flight 15 around the SUN facility. Mass emission rates have been determined for this facility using the TERRA algorithm, where elevated and surface emissions can be quantified separately. The mean hourly CO_2_ emission rates (t h^−1^) during all flights covering the four major surface mining operations in the oil sands are shown in the inset table. Uncertainties represent the standard error $\left( {\frac{\sigma }{{\sqrt N }}} \right)$ for the multiple flights over a facility. Map data: Google, Image Landsat, CNES/Airbus, Digital Globe 2017. Source data for the inset table are provided in Supplementary Data 1

Thirteen aircraft flights around the OS surface mining facilities in Alberta (Supplementary Table 1) were conducted in the summer of 2013, with virtual polygon flight boxes surrounding a given facility that encompassed all CO_2_ emission sources, including mining, processing, upgrading, and tailings release^17^ (Supplementary Fig. 1a–c). Approximately 5–12 different altitudes were flown for each box, depending upon the facility. Background subtracted CO_2_ data from these flights were used as inputs into a Top–down Emissions Rate Retrieval Algorithm (TERRA)^18^ (see Methods), from which hourly CO_2_ emission rates were derived. For the OS surface mining facilities, an example virtual box is shown in Fig. 1 for the Suncor (SUN) facility and the resultant hourly CO_2_ emissions for all facilities in the inset table. The downwind side of the virtual box of Fig. 1 demonstrates the complexity of CO_2_ plumes from this facility including emissions from both ground sources and elevated stacks, which can be quantified separately. Based on these flights, the largest hourly CO_2_ emissions are associated with Syncrude Mildred Lake (SML; 1650 ± 134 t h^−1^) and SUN (1220 ± 138 t h^−1^) facilities, followed by Canadian National Resources Ltd Horizon (CNRL; 538 ± 68 t h^−1^) and Shell Albian and Jackpine (SAJ, now part of CNRL, 423 ± 55 t h^−1^), all of which are dominant compared to CO_2_ from local transportation within the virtual box (see Supplementary Note 1). Propagated uncertainties (see Methods) associated with hourly emission rates for single flights were small (≈8–26%; Supplementary Table 1), with emission rates consistent between flights (relative standard error (RSE) ≈ 8–13%; Fig. 1) despite being conducted on various days spanning approximately 1 month.

### Emission intensities

**Fig. 2:**
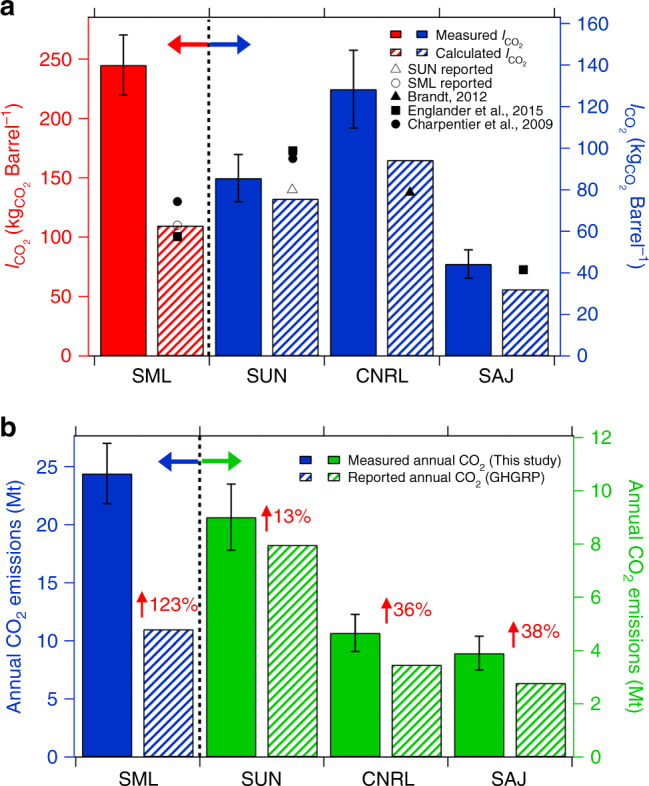
CO_2_ emission intensities and total emissions. **a** Aircraft measurement derived (mean) and calculated well to the refinery gate CO_2_ emission intensities $(I_{{\mathrm{CO}}_2})$ for the 4 major surface mining operations in the oil sands in 2013 (*n* *=* 3–6). Calculated emission intensities use publically available data from the GHG reporting program (GHGRP) for the same facilities (2013). Opaque data points represent facility-specific model calculation of $I_{{\mathrm{CO}}_{2{\mathrm{eq}}}}$ in the literature and open symbols represent $I_{{\mathrm{CO}}_{2{\mathrm{eq}}}}$ reported directly by the industry for 2013. SAJ (now part of CNRL) does not upgrade to synthetic crude oil on site, hence its intensity is per barrel of bitumen. The $I_{{\mathrm{CO}}_2}$ for SML includes emissions from SAU. **b** Annual emissions of CO_2_ measured for the four major surface mining facilities in 2013 compared to those reported by industry to the GHGRP. Error bars in  **a**, **b** represent the propagated uncertainty of the mean value. Source data are provided in Supplementary Data 1

For each surface mining facility, the CO_2_ emission intensity $( {I_{{\mathrm{CO}}_2}} )$, in units of ${\mathrm{kg}}_{{\mathrm{CO}}_2}{\mathrm{barrel}}^{ - 1}$ of synthetic crude oil (SCO), is shown in Fig. 2a. The $I_{{\mathrm{CO}}_2}$ is derived by upscaling the hourly CO_2_ emissions (Fig. 1, Supplementary Table 1) to monthly values (see Methods) and dividing by known monthly facility production volumes of SCO^19^. The overall lifecycle GHG emission intensity for any petroleum source is dominated by the combustion of its end product (e.g., gasoline), which is relatively constant and accounts for 70–80% of the total GHG emission^20^. Hence, to compare the GHG emission across various petroleum sources, estimates typically only include processes occurring from the well to the refinery gate (WTRG) and are based on quantities derived from various emission models^21–23^. The current results are the first top–down WTRG estimates of $I_{{\mathrm{CO}}_{2}}$ for the OS using direct CO_2_ measurements, thus providing a benchmark for the assessment of previous model estimates of $I_{{\mathrm{CO}}_{2{\mathrm{eq}}}}$ (which include CH_4_). Such model estimates are known to be highly variable, due mainly to the types of sources included in the model, uncertainties in fuel-related assumptions, and other methodological challenges^20,24–26^. The derived $I_{{\mathrm{CO}}_2}$ here ranged from 44.3 ± 6.8 to 245 ± 25.4 ${\mathrm{kg}}_{{\mathrm{CO}}_2}{\mathrm{barrel}}^{ - 1}$ (Fig. 2a and Supplementary Table 2) for the 4 major surface mining facilities in operation in 2013. Including measurement-based CH_4_ emissions reported previously^12^ in the present CO_2_ emissions results in $I_{{\mathrm{CO}}_{2{\mathrm{eq}}}}$ ranging from 47.6 ± 7.6 to 267 ± 31 ${\mathrm{kg}}_{{\mathrm{CO}}_{2{\mathrm{eq}}}}{\mathrm{barrel}}^{ - 1}$, which is dominated by the contribution from CO_2_ (see Methods). With the exception of SML (which includes emissions of SAU), the measured $I_{{\mathrm{CO}}_{2{\mathrm{eq}}}}$ are somewhat larger than the $I_{{\mathrm{CO}}_{2{\mathrm{eq}}}}$ calculated using overall industry average inputs from up to 6 years earlier (77–122 ${\mathrm{kg}}_{{\mathrm{CO}}_2}{\mathrm{barrel}}^{ - 1}$ SCO)^25^. However, the measured $I_{{\mathrm{CO}}_{2{\mathrm{eq}}}}$here does not include the emissions associated with transport of SCO to the refinery gate. Including such emissions would make the actual $I_{{\mathrm{CO}}_{2{\mathrm{eq}}}}$ even larger than industry average model values^20,25,26^. The difference between estimated and reported intensities is even larger when comparing the currently estimated $I_{{\mathrm{CO}}_2}$ (or $I_{{\mathrm{CO}}_{2{\mathrm{eq}}}}$) with those reported for specific facilities in the same year (2013). In this case, the estimated $I_{{\mathrm{CO}}_2}$ are 13–123% larger than those calculated using CO_2_ emissions reported to Environment and Climate Change Canada’s GHG reporting program (GHGRP)^15^ and reported production volumes^19^ (see Methods). The measurement-based $I_{{\mathrm{CO}}_2}$ values are similarly larger than the $I_{{\mathrm{CO}}_{2{\mathrm{eq}}}}$ provided directly by industry^27,28^ for 2013 (Fig. 2a). The estimated $I_{{\mathrm{CO}}_2}$ for SUN is in reasonable agreement with the calculated estimates shown in Fig. 2a, being only 13% higher and within the measurement uncertainty. In contrast, estimates of the mean $I_{{\mathrm{CO}}_2}$ for CNRL, SAJ, and SML/SAU are 36%, 38%, and 123% greater than those calculated using publicly available data^15,19^ and larger than the facility-specific $I_{{\mathrm{CO}}_{2{\mathrm{eq}}}}$ modeled in recent studies^24–26^.

### Annual emission estimates

Based upon the aircraft-estimated $I_{{\mathrm{CO}}_2}$, the annual CO_2_ emissions for each facility are estimated (see Methods) and shown in Fig. 2b, together with the CO_2_ emissions reported by industry to the GHGRP for 2013. The aircraft measurement-based annual CO_2_ emissions from the 4 major OS facilities ranged from 3.9 ± 0.6 to 25 ± 3 Mt. These emissions are higher than the reported CO_2_ emissions, reflecting the differences in the aircraft measurement-estimated vs facility-estimated $I_{{\mathrm{CO}}_2}$, with the largest statistically significant difference (see Methods) of 13 Mt year^−1^ for SML/SAU (123%) and the smallest for SUN (13%).

**Fig. 3:**
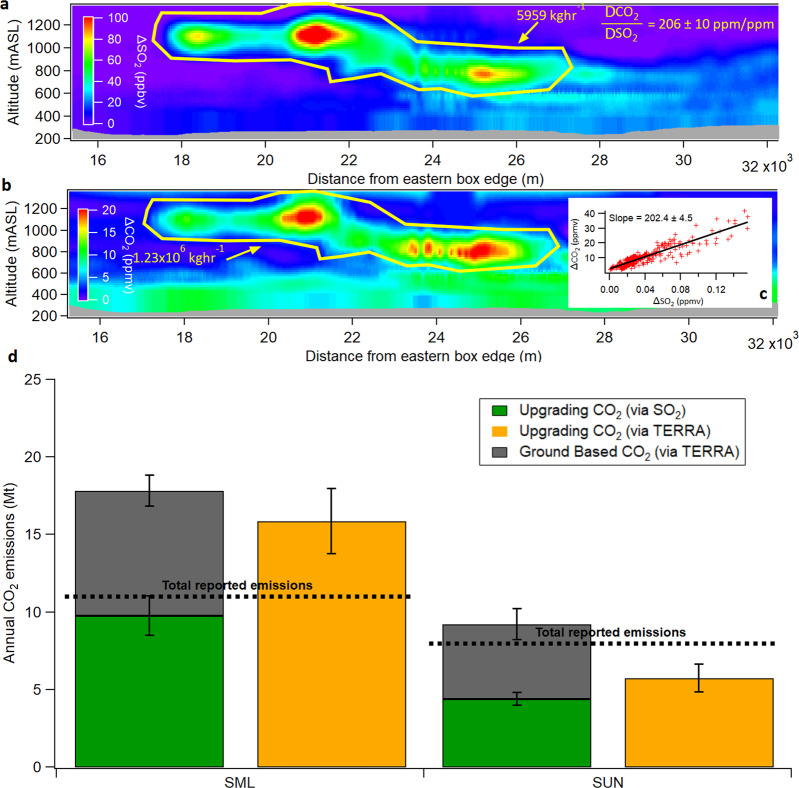
Emissions of CO_2_ for upgrading operations and ground-based sources in the oil sands. **a** Background subtracted SO_2_ in elevated plumes from OS upgrading emissions during flight 18 derived with TERRA. **b** Background subtracted CO_2_ in the same elevated plumes of flight 18. **c** Direct correlation between background subtracted CO_2_ and SO_2_ within plumes. **d** Annual emissions of CO_2_ from upgrading stacks for SML and SUN derived using the CO_2_:SO_2_ emission ratios measured in plume (green bars; see Methods). Orange and gray bars represent upgrading and ground-based emissions, respectively, derived using TERRA directly and up-scaled as described in Methods. Dashed lines represent total facility-reported CO_2_ emissions to the GHG reporting program. Error bars in Fig. 2a, b represent the propagated uncertainty of the mean value. Source data are provided in Supplementary Data 1

The differences between aircraft-estimated and facility-reported CO_2_ emissions for SML and SUN are further validated through a separate approach using measurements of sulfur dioxide (SO_2_) and reported mined OS ore volumes^19^ as tracers for stack and ground-based CO_2_ emissions, respectively (see Methods). For these two facilities, the emissions of SO_2_ are associated with bitumen upgrading and are generally confined to elevated stack outflows only, leading to elevated SO_2_ plumes downwind (Fig. 3a, Supplementary Fig. 2). Hence, CO_2_ plumes from both these facilities are clearly split into an elevated stack plume that is closely associated with the SO_2_ plume and a ground-based plume (Fig. 3b). Multiplying the CO_2_ to SO_2_ emission ratios in the elevated plumes (Fig. 3a, c) with measured annual SO_2_ emissions^29^ provides an alternative means to determine annual stack CO_2_ emissions specifically (Fig. 3d) (See Methods and Supplementary Note 2). Similarly, a ground-based emission ratio was estimated by normalizing the TERRA-derived ground-based emissions by the monthly volume of mined OS ore (see Methods). Using these approaches, the separated annual CO_2_ emissions from stacks and ground emissions at SML are estimated at 14.2 ± 1.3 and 8.1 ± 1.0 Mt, respectively (Fig. 3d). This result is consistent with that derived using TERRA specifically for elevated plumes (Fig. 3d) and indicates that stack CO_2_ emissions alone are ~29% greater than the reported facility total CO_2_ emissions for SML/SAU (all sources; Fig. 3d). Furthermore, the combined emissions (stacks + ground based; 22.3 ± 3.5 Mt) for SML are in agreement with the facility total derived above through the use of $I_{{\mathrm{CO}}_2}$ (24.5 ± 3.0 Mt, which includes SAU; Fig. 2b), supporting the discrepancy between total aircraft measurement-based estimated and total reported CO_2_ emissions for this facility (Fig. 2b). In contrast, stack CO_2_ emissions estimated for SUN are ~20% lower than the reported facility total CO_2_ emissions (all sources; Fig. 3d), while the total measured stack (6.4 ± 0.4 Mt)+ground CO_2_ emissions (4.8 ± 0.7 Mt) are only slightly higher than the reported CO_2_ emissions of Fig. 2b. Based on Fig. 3d, it is estimated that stack CO_2_ emissions for SML and SUN account for 61 ± 12% and 67 ± 13% of their respective total emissions, in relative agreement with available model estimates^20,24^.

**Fig. 4:**
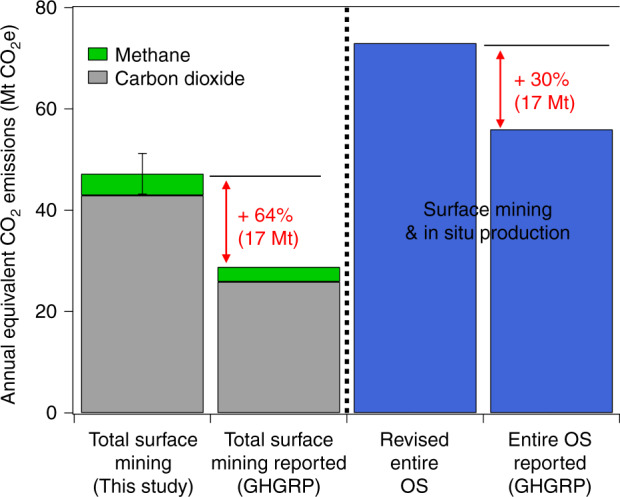
Total oil sands (OS) annual greenhouse gas (GHG) emissions compared to those reported to the GHG reporting program. Methane emissions from Baray et al.^12^ are added to the surface mining CO_2_ measured here, to be comparable to reported ${\mathrm{CO}}_2{\mathrm{e}}$. A global warming potential (GWP) of 25 is used for methane. Blue bars include in-surface and in situ OS extraction. Revised estimate for total OS emissions includes the measured surface mining emissions determined in this study and the reported emissions from in situ operations. Errors associated with annual CO_2_ (Fig. 2b) and CH_4_ estimates have been added in quadrature: $\delta = \sqrt {\delta _{{\mathrm{CO}}_2}^2 + \delta _{{\mathrm{CH}}_4}^2}.$ Source data are provided in Supplementary Data 1

The overall impact of the differences between measured and reported GHG emissions found here is large. Put into context, the unaccounted emissions represent a total CO_2_e (100-year GWP timescale) emission increase of ~64% (or 17 Mt year^−1^) over that reported for OS surface mining operations (Fig. 4) and a ~30% increase for the entire OS (including reported in situ emissions) (Fig. 4). Such an absolute emission quantity (17 Mt year^−1^ CO_2_e) is similar to the GHG emissions from a metropolitan area the size of Seattle^30^ or Toronto^31^. Additionally, GHG emission reports from OS in situ extraction facilities have yet to be evaluated using atmospheric measurements. This suggests that the potential exists for further unaccounted GHG emissions, as partially demonstrated by Lagrangian flights that show hourly CO_2_ emissions downwind that are larger than the sum of individual facility emission rates (see Supplementary Note 3).

## Discussion

There are important implications associated with the current findings. Since many stationary combustion sources in the O&G sector derive their emissions using similar Tier 1–3 protocols^5^, this issue may not be unique to the OS or to CO_2_. Indeed, the Tier 3 approach (and other provincial reporting regulations) used for the OS is considered to utilize the best possible data, and yet top–down and bottom–up results here remain significantly different. This may suggest that other GHG sources or industries in other regions, which use less accurate bottom–up methods (i.e., Tier 1) could exhibit similar discrepancies between top–down and bottom–up estimates. The results here further suggest that top–down emission estimates can be used to inform the development of federal and provincial emission regulations and highlights the value of atmospheric measurements in tracking and/or monitoring progress in terms of emission reduction targets. Equally important is that the underlying data used in reporting to the GHGRP are also used in formulating OS GHG emission estimates in Canada’s NIR to the UNFCCC^16^. Both the GHG emission estimates in the GHGRP and NIR are considered Tier 3 according to the IPCC as they use the best available information specific to the industry and provide the highest possible accuracy^5,16^. As a result, the GHGRP and NIR emission data vary little from each other for specific facilities, with uncertainties associated with OS fugitive CO_2_ emissions^16^, overall emissions for the Canadian energy sector^16^, and stationary combustion emission factors^5^ reported as approximately ±6%, ±3%, and ±< 10%, respectively. However, the top–down measurement results here indicate that the total inventory GHG estimates for the OS sector may need to be revised upward by at least 30%. Most importantly, the results imply that complex emission factors and activity data used in NIRs should be periodically re-assessed and highlights the potential need for new IPCC guidelines for atmospheric measurement-based validation of emissions. Such guidelines are particularly important since atmospheric measurements are now recognized for their ability to systematically monitor GHG emissions in support of the Paris Agreement^32^. Finally, this type of atmospheric observation can directly inform stakeholders at the subnational scale about unknown or poorly quantified GHG sources at the facility level, as a contribution to the recently adopted integrated global GHG Information System^33^ of the World Meteorological Organization and United Nations Environment Program.

## Methods

### Aircraft measurements

Aircraft measurements of CO_2_ and CH_4_ over the Athabasca OS region of northern Alberta were performed from August 13 to September 7, 2013 as part of an intensive field campaign. Details of the study objectives, aircraft implementation, and other technical aspects have been described previously^17,34^. During the study, 22 flights totaling 84 h were conducted over individual facilities of the OS and downwind of the facilities at distances up to 120 km. The flight patterns have been shown previously^17^, 13 of which were used to quantify primary emissions from individual OS facilities, and 4 conducted to quantify their transformation downwind. Emission quantification flights were conducted by flying a 4- or 5-sided polygon, at multiple altitudes, resulting in 21 separate virtual boxes encompassing 7 OS facilities. Results from 17 flights were used in the current analysis, while the others were excluded because of highly variable wind speeds and direction and/or the presence of confounding pollutant interferences from adjacent OS facilities. The specific facilities studied and their corresponding flight numbers are given in Supplementary Table 1. Three of the flights (F7, F19, and F20) examined the photo-chemical transformation of pollutants downwind of the OS facilities^34^ and are used here to quantify total emissions from all OS surface mining facilities (for comparison to the sum of individual emission flights), as they encompass the majority of emissions from the region. These transformation flights were designed as Lagrangian, such that the same air parcel in the OS plumes was sampled successively, 1 h apart, in virtual screens constructed from level flight tracks at multiple altitudes (Supplementary Fig. 3, Supplementary Table 1). There were no industrial emissions between the screens of each flight.

Gas, particle, meteorological, and aircraft state measurements were made during flights, with a detailed description provided elsewhere^17,34,35^. The current work makes use of a subset of these measurements specifically for the quantitation of CO_2_ and SO_2_ gases. CO_2_ was measured using a Picarro model G2401-*m* instrument (http://www.picarro.com/products_solutions/gas_analyzers/flight_co_co2_ch4_h2o) with a time resolution of 2 s^12^. The instrument sampled ambient air through a 6.35-mm (1/4 inch) diameter perfluoroalkoxy, backwards facing sampling line with a residence time of ~2 s. Calibrations were performed before, during, and after the field campaign using a range of mixing ratios from NIST certified standard cylinders (350–450 ppm). The precision of the CO_2_ measurements in flight were estimated to be <100 ppb. SO_2_ was measured using a Thermo Scientific 43iTLE analyzer with a time resolution of 1 s. The SO_2_ instrument was calibrated with NIST certified standard cylinders over a range of 0–400 ppb. The standard deviation of the calibration slopes was 0.9% and the average standard deviation of the 1-s data during calibrations was 1.99 ppb^18^. The time delay for the SO_2_ measurement, due to the gas traversing the length of the inlet, was ≈6 s and was corrected for in the data. NO and NO_2_ measurements were made using two Thermo Scientific 42iTLE analyzers at 1 Hz (NO_*X*_ averaged to 2 s) with a sample residence time of ≈3–4 s. Each instrument was calibrated with NIST standard cylinders of NO. High frequency wind data were averaged to 1 or 2 s for subsequent analysis in the emission rate algorithm.

### Top–down emission rate retrieval algorithm analysis

CO_2_ emission rates for each OS surface mining facility and for the entire OS region were derived using an algorithm designed to estimate pollutant transfer rates through the virtual boxes/screens of aircraft measurements. The algorithm is based on the Divergence Theorem to resolve mass balance (TERRA)^18^ and has been used extensively for determining emission rates of volatile organic compounds (VOCs), black carbon, CH_4_, secondary organic aerosol (SOA), organic/inorganic acids, and SO_2_^17,18,34,36^ Emissions are derived with the TERRA algorithm by combining box-like aircraft flight patterns with pollutant concentration measurements at high time resolution and wind speed and direction data. The algorithm applies simple kriging^18^ to the data to produce virtual walls of gridded 20 × 40 m^2^ mesh on the virtual box, resolves the air mass balance within the virtual box, and determines the mass transfer rates across the walls (including the top of the box) to derive a net emission rate for a pollutant. In the case of virtual screens (rather than boxes), TERRA quantifies the mass transfer rate of a pollutant (kg h^−1^) through the virtual screens, in the same manner as it does for a single wall of a virtual box flight^34,36^.

The use of the standard TERRA to estimate emissions accounts for incoming and outgoing air mass advective and turbulent fluxes within a volume, including the top of the virtual box, and is ideal for species that have little background contribution to the measured concentration. However, in the case of CO_2_ that has a large background concentration, this approach can introduce large uncertainties if even a small portion of the measured CO_2_ is calculated to leave through the top of the box. To address this uncertainty, a background CO_2_ is subtracted prior to the use of TERRA, such that the transfer rate through any one outgoing side of a box (or single screen) represents the facility emission rate. This approach has been used successfully for CH_4_^12^, VOCs^35,36^, and SOAs^34^ and is similar to other single screen approaches^37,38^. Background CO_2_ was determined using upwind measurements for box flights and screen flights (where available). For screen flights where upwind measurements were not conducted, the levels at the edges of the screen were used as background. The background between upwind data was linearly interpolated and box-car smoothed within a 20–40-min moving window to derive a variable baseline CO_2_ for the entire 2–3 h flight. Examples of the derived CO_2_ baseline are shown in Supplementary Fig. 3. The uncertainty added to the final emission rate for each flight contributed by the baseline estimation method was examined by varying the derived CO_2_ baseline by the standard deviation of the upwind data (±0.5–3 ppm CO_2_), followed by input into TERRA. This baseline CO_2_ sensitivity analysis resulted in CO_2_ emission rates that varied by 1–33% (*δ*_B_; Supplementary Table 1). This relatively small variation in emission rates caused by the background CO_2_ is due to the short duration of the flights, which were flown during an established planetary boundary layer and because there was significant enhancement of plume CO_2_ of up to >80 ppm above background. In general, a changing boundary layer depth could in principle introduce additional uncertainty in the emission results from TERRA. However, the flights were intentionally conducted during mid-afternoon hours, when the boundary layer was constant. This was assessed through vertical profiles performed before and after the emission estimation portions of the flights. Under conditions where boundary layer depth was stable, real-time vertical profile data (pollutants, water vapor, temperature) from the flights were used to ensure that the highest flight track was always above the top of the boundary layer, hence ensuring that the entire plume from the various facilities was contained within the specific flight box or within the flight screen.

It has been demonstrated previously that extrapolation of pollutant concentrations from the lowest flight level to the ground is the main source of uncertainty in TERRA, with an overall uncertainty in the derived emission rates of approximately 20%^18^. This was determined previously^18^ through a sensitivity analysis in a series of closed-loop numerical experiments. In such experiments, a simulated Gaussian plume driven by a known emission flux was created and sampled with the TERRA algorithm, while also using various forms of extrapolation to the ground (i.e., linear to the ground, exponential, constant, linear to zero). A similar analysis was performed in the current study, with the differences in extrapolations also resulting in a <20% difference in final estimated emissions. For pollutant sources that are entirely above the lowest flight level, extrapolation to the ground is not relevant and the associated uncertainty in the TERRA-derived emissions has been demonstrated to be ≈ 4%^18^. This was determined by comparing the TERRA-derived emissions of SO_2_ with continuous emissions monitoring system (CEMS) data for the same facility stack (CNRL) and for the exact hour of the flight. We note that CEMS data are regularly audited and mandated to be accurate to within 10%. Further comparisons of TERRA-derived SO_2_ stack emission rates with those concurrently measured with CEMS indicate that this uncertainty may be even smaller (<1%) as shown in Supplementary Fig. 4a. However, the larger 4% uncertainty for elevated stack emissions is used in this analysis. The excellent agreement between TERRA emissions and the audited CEMS data provide confidence that uncertainties associated with possible under sampling are negligible. The uncertainties for stack and ground-based emissions (i.e., 4% and 20%) are applied separately in cases where low level and elevated stack sources are clearly spatially differentiated, which are further propagated with the uncertainties associated with the background CO_2_ determination described above. The overall propagated uncertainty associated with the hourly emission rates derived for individual facilities ranged from 8% to 28% $(\Delta E_{{\mathrm{CO}}_2})$ for facility flights and from 24% to 38% for downwind transformation flights as shown in Supplementary Table 1. In contrast to the emission box flights, the largest uncertainty in the transfer rates through the downwind screens arises from the background subtraction of CO_2_ (*δ*_B_ in Eq. 9 below) due to the higher variability in background CO_2_ over a typically longer flight time. Such uncertainties are included in the emission results from TERRA and reported separately in Supplementary Table 1.

### Upscaling OS emissions

The hourly emissions of CO_2_ derived with TERRA in Fig. 1 and Supplementary Table 1 are used to calculate the CO_2_ emission intensity $\left( {I_{{\mathrm{CO}}_2}} \right)$ for individual facilities (${\mathrm{kg}}_{{\mathrm{CO}}_2}{\mathrm{barrel}}^{ - 1}$ SCO) as a first step to estimating annual CO_2_ emissions. This emission intensity is derived as

1 $$I_{{\mathrm{CO}}_2} = \frac{{E_{{\mathrm{M}}_{{\mathrm{CO}}_2}}}}{{P_{{\mathrm{SCO}}}}}$$

where $E_{{\mathrm{M}}_{{\mathrm{CO}}_2}}$ is the measured emission rate of CO_2_ for a given facility scaled to a month and *P*_SCO_ is the reported monthly production volume of SCO in units of barrels (reported to the Alberta Energy Regulator (AER)). Since production volumes are reported as monthly totals^19^, the measured hourly emission rates ($E_{{\mathrm{CO}}_2}$) must be scaled up to a month to be used in the above equation (see below). The fact that the relative standard deviation between flights for a single facility spanning a period of a month was <10% supports this monthly upscaling of emission rates. Alternatively, production volumes and hourly CO_2_ emissions can both be scaled to daily values. For simplicity, we have chosen to only scale the emissions measurements to monthly values; however, scaling both the emissions up and the production down to 1 day results in the same CO_2_ intensities (within <1%).

Scaling the hourly CO_2_ emissions to daily or monthly values with a simple 24 h (i.e., 1 day) or 744 h (i.e., 1 month) scaling factor may overestimate the total emissions during that time period. Rather, we have used an approach described previously for VOC emissions^17^. As described in Li et al., the TERRA-derived hourly emission rates are converted to daily (or monthly) emission rates based on the diurnal (or monthly) changes in bitumen production. This approach is ideally suited to CO_2_ as it is not likely to have the added complexity of temporally/temperature-dependent emissions from non-combustion sources, which may not scale with production. For combustion-based emissions, the hourly emission rate (*E*_m_) is related to the average hourly emission rate ($\overline {E_{\mathrm{m}}}$), the hourly bitumen production *q* at the hour of measurement, and the 24-h production *p*.

2 $$E_{\mathrm{m}} = 24 \ast \overline {E_{\mathrm{m}}} \left( {\frac{q}{p}} \right)$$

Since combustion and thus production is accompanied by NO_*X*_ emissions, the ratio *q*/*p* may be derived as

3 $$\left( {\frac{q}{p}} \right) = \frac{{E_{{\mathrm{NO}}_X}}}{{\mathop {\sum }\nolimits E_{{\mathrm{NO}}_X}}}$$

where $E_{{\mathrm{NO}}_X}$ is the hourly emission rate during aircraft measurement, and $\mathop {\sum }\nolimits E_{{\mathrm{NO}}_X}$ is the daily NO_*X*_ emission rate. Thus a factor *C*_daily_, for converting hourly to daily emission rates, can be expressed as

4 $$C_{{\mathrm{daily}}} = 24 \ast \frac{q}{p} = \frac{{24 \ast E_{{\mathrm{NO}}_X}}}{{\mathop {\sum }\nolimits E_{{\mathrm{NO}}_X}}}$$

Similarly, a scaling factor to convert hourly to monthly emission rates is derived as

5 $$C_{{\mathrm{monthly}}} = \frac{{744 \ast E_{{\mathrm{NO}}_{\mathrm{X}}}}}{{\mathop {\sum }\nolimits E_{{\mathrm{NO}}_{\mathrm{X}}}}}$$

where ${\sum} {E_{{\mathrm{NO}}_X}}$ is the monthly emission rate. Using NO_*X*_ as an indicator of production is convenient since hourly, daily, and monthly NO_*X*_ emission rates are available from CEMS measurements. The underlying assumption is that both CO_2_ and NO_*X*_ emissions are proportional to SCO production, which is consistent with the fundamental assumptions for emission factor-based emission reporting, and consistent with the empirical relationship between CO_2_ and NO_*X*_ observed here (Supplementary Fig. 4b). Although various in-plant sources of combustion exist, each with a different NO_*X*_/CO_2_ ratio, this assumption requires the aggregate NO_*X*_ emissions (sum of all CEMS data) to vary with production. While SO_2_ was chosen previously as a surrogate for production^17^, we have chosen to use NO_*X*_, as the CEMS data for NO_*X*_ is more likely to include other stack combustion sources in addition to the upgrader stacks, which are the main source of SO_2_. While it is not known whether the NO_X_/CO_2_ ratios from different sources within a facility are temporally invariant, the standard deviation of the slope of the empirical relationships in Supplementary Fig. 4b are used as a measure of the potential error introduced by variable NO_*X*_/CO_2_ ratios during the study (*δ*_V_). The CEMS data for $E_{{\mathrm{NO}}_X}$ from the facilities are reported to be accurate to within 10%^39^ (*δ*_CEM_). The uncertainty in each *C*_monthly_ scaling factor per flight (*δ*_C_) is then derived as

6 $$\delta _{\mathrm{C}} = \sqrt {2(\delta _{{\mathrm{CEM}}}^2) + \delta _{\mathrm{V}}^2}$$

and ranged from ≈14% to 15%. The mean scaling factor *C*_monthly_ is derived to be 0.83 ± 0.05, 1.00 ± 0.07, and 0.89 ± 0.06 for SML, SUN, and CNRL, respectively. For other facilities where no CEMS data were available, the average of the terms above is used to calculate *C*_monthly_ (0.91 ± 0.13). The monthly CO_2_ emission rates ($E_{{\mathrm{M}}_{{\mathrm{CO}}_2}}$) for a given facility are then derived as

7 $$E_{{\mathrm{M}}_{{\mathrm{CO}}_2}} = E_{{\mathrm{CO}}_2} \times \frac{{744}}{{C_{{\mathrm{monthly}}}}}$$

where $E_{{\mathrm{CO}}_2}$ is the TERRA-derived hourly emission rate (720/*C*_monthly_ is used for September)*.* This is subsequently used to compute the CO_2_ emission intensities $( {I_{{\mathrm{CO}}_2}} )$ via Eq. (1) above and shown in Fig. 2a and Supplementary Table 2.

### Annual OS CO_2_ estimates and uncertainties

Annual CO_2_ emissions (*E*_annual_) for individual facilities are derived by multiplying the top–down derived CO_2_ emission intensities (Supplementary Table 2), by the reported annual SCO production volumes for the year 2013^19^:

8 $$E_{{\mathrm{annual}}} = I_{{\mathrm{CO}}_2} \times P_{{\mathrm{SCO}}_{{\mathrm{annual}}}}$$

The derived annual CO_2_ emissions have an overall relative error (*δ*_E_) with contributions from the errors associated with the application of TERRA (*δ*_T_ ≈ 7–20%), the baseline CO_2_ estimation (*δ*_B_ ≈ 1–20%), the hourly to monthly scaling factor (*δ*_C_ ≈ 14–15%; above), the relative measured variability in TERRA-derived emissions from multiple flights per facility (standard error; *δ*_D_ ≈ 4–10%), and the reported production volumes of SCO (*δ*_P_ ≈ 1%). The overall relative uncertainty in the annual emission rate (*δ*_E_ = *ΔE*_annual_/*E*_annual_) can hence be calculated as

9 $${\delta} _{\mathrm{E}} = \sqrt {{\delta} _{\mathrm{T}}^2 + {\delta} _{\mathrm{B}}^2 + {\delta} _{\mathrm{C}}^2 + {\delta} _{\mathrm{D}}^2 + {\delta} _{\mathrm{P}}^2}$$

for each flight and ranged from 17% to 32% depending on the facility (Supplementary Table 2). The mean annual emissions (across individual flights) for each facility is shown in Fig. 2b, with the error bars representing the propagated uncertainty of the mean (see Supplementary Table 2).

The uncertainty associated with the calculated emission intensities $( {I_{{\mathrm{CO}}_2}} )$ of Fig. 2a are also derived with Eq. (9), but using a value of *δ*_P_ (≈ 1%) for monthly reported production (rather than annual production). This also results in a calculated uncertainty in $I_{{\mathrm{CO}}_2}$ of 17–32%. The absolute uncertainties in $I_{{\mathrm{CO}}_2}$ derived for each individual facility flight are provided in Supplementary Table 2 and ranged from ±10 to ±76 kg CO_2_ barrel^−1^, with a propagated error in the mean $I_{{\mathrm{CO}}_2}$ for each facility ranging from ±7 to ±26 kg CO_2_ barrel^−1^ (see Supplementary Table 2). It is expected that applying the emission intensities $( {I_{{\mathrm{CO}}_2}} )$ derived for 1 month in this study across the entire year to determine annual emissions is significantly more accurate than attempting to scale the measured CO_2_ emissions from these flights directly to a full year. This intrinsically assumes that the measured emission rates are more representative of a month than they are to a full year and only requires $( {I_{{\mathrm{CO}}_2}} )$ to be relatively constant from month to month (see other considerations and assumptions section).

Annual CH_4_ emissions used in Fig. 4 were taken directly from previous work^12^. Deriving an emission intensity for methane (to be included in the total $I_{{\mathrm{CO}}_{2{\mathrm{eq}}}}$) using similar upscaling factors as was done for CO_2_ may not be fully appropriate for CH_4_, as most CH_4_ in the OS is not derived via combustion. Hence $I_{{\mathrm{CO}}_{2{\mathrm{eq}}}}$ for CH_4_ specifically was derived by dividing previously reported annual CH_4_^12^ by annual production volumes of SCO^19^. Regardless, applying the upscaling approach for CO_2_ described above to hourly CH_4_ resulted in CH_4_ emission intensities that differed by <5% and remain a small contributor to overall OS GHG emissions (using 100-year GWP).

The statistical significance of the differences between reported annual CO_2_ emissions and those derived here (Fig. 2b) are investigated through the use of a single-sample *t* test, since as with all reported emissions there is only a single reported value for each facility. This analysis tests the null hypothesis that can be stated as: $\bar M$ = *μ*_o_, i.e., the mean of all aircraft measurement results for a given facility ($\bar M$) equals the reported value (*μ*_o_). Consequently, the *t* statistic can be given as:

10 $$t = \frac{{\bar M - \mu _{\mathrm{o}}}}{{\sqrt {\frac{{{\sum} {{{X}}^2} - \left( {\left( {\left( {{\sum} {{X}} } \right)^2} \right)/N} \right)}}{{\left( {N - 1} \right)N}}} }}$$

where $\bar M$ is the sample mean, *μ*_o_ is the reported value, *X* are the aircraft-based emission rates from all flights of a facility, and *N* is the number of flights for each facility, with a degree of freedom of *N* − 1. Using this test for the SML facility results in a *t* = 8.4, which translates into a *p* value of 0.0004. This indicates that the aircraft measurement-based emission results for SML are statistically and significantly different from the reported emission at the 99.9% confidence level. The results for other facilities resulted in *t* values of 1.95, 2.32, and 2.69 for SUN, CNRL, and SAJ, respectively. This corresponds to means that are different than reported values at *p* < 0.2, <0.1 and <0.1 (i.e., ~80–90% confidence level).

### Other considerations and assumptions

There are potentially additional considerations that should be noted with respect to the estimated emission intensities and annual emissions particularly when compared to traditional bottom–up approaches. Such bottom–up approaches rely upon accurate and detailed knowledge of: the technology in use, emission factors, fuel mixture data, operating conditions, quality of maintenance, control technologies in use, carbon content data, and the lower or higher heating values (LHV vs HHV) of the process. These items can have a degree of uncertainty associated with them and do not include the potential for unintentional errors or omissions of sources (among the very many bottom–up sources that exist). While aircraft measurements of CO_2_ concentrations and associated top–down methodologies are also subject to a range of quantifiable uncertainties, the approach provides a useful and complimentary dataset to bottom–up methods, which can identify when further investigation into emissions is required, particularly when discrepancies exist. While it is not possible to unequivocally determine the root causes of the observed discrepancies here, it is speculated that that they may arise from a combination of factors, including outdated emission factors and activity data, unknown/poorly updated fuel carbon content data, poorly quantified area sources, and human error. Such difficulties are expected to be faced by various O&G and other industries, suggesting the potential for universal differences between top–down and bottom–up emission estimation methods.

Some additional uncertainties for the top–down methodology (those not already stated in methods) are also possible. For example, the aircraft measurements were performed over the course of a month and appropriately scaled, taking into account monthly production to derive emission intensity. A seasonal change in emission intensity during non-sampled months is possible, although we expect that this should be minimal (<15%) as noted previously^26^, particularly since seasonal emissions of CO_2_ from fugitive sources such as tailing ponds are small contributors to overall CO_2_ emissions^40^. Since much of the CO_2_ is a result of combustion, it expected that increased energy/heat would be required during the winter, potentially resulting in increased emission intensities compared to the summer. In addition, changes in the fuel mixture used within facilities over the year can influence the derived emission intensities. For example, increased use of pet coke as fuel (which is known to emit more CO_2_) during the month of this study could bias the derived emission intensities high compared to other months. The underlying assumption in this regard is that the relative proportion of fuel types used during the aircraft flights was similar to other months. This is supported by OS plant statistics^19^, which show that the fraction of pet coke used varied by <10% month to month in 2013 for SML. Regardless, the carbon content of pet coke is only 10–15% higher than that of natural gas and process gas used for fuel and would only apply to 60–70% of total facility emissions (i.e., not from the mining process).

Other end products arising from OS operation (i.e., not SCO) are also possible, which could introduce some uncertainty into the emission intensity estimates that use SCO production in the denominator of the equation for $I_{{\mathrm{CO}}_2}$. This is also expected to be relatively small as SML and SUN generally produce ~90% SCO^26^. Regardless, using an alternative emission factor approach (i.e., CO_2_/SO_2_ or CO_2_/ore mined) results in very similar annual emissions compared to the emission intensity approach. The results from these independent approaches are good evidence that they are valid and support the underlying assumption that NO_*X*_ emissions on aggregate are proportional to SCO production (see Methods). Regardless, provided that the product mixture does not change significantly from month to month, the derived $I_{{\mathrm{CO}}_2}$ remains a valid emission factor that can be used to calculate annual emissions. The OS plant statistics^19^ support the assumption of a constant product mixture as it varies by <10% month to month. Finally, the possibility of unforeseen facility maintenance, open burning of overburden, or increased flaring resulting in increased CO_2_ emissions cannot be ruled out. However, there was no evidence of maintenance or burning occurring during this study, and flaring is expected to account for a very small fraction of the overall CO_2_ emissions. Regardless, such events are unlikely to have occurred systematically across the exact days of study during the months of August and September.

### Reporting summary

Further information on experimental design is available in the Nature Research Reporting Summary linked to this article.

## Supplementary information

Supplementary Information

Reporting Summary

## Data Availability

All data used in this publication are freely available on the Canada-Alberta Oil Sands Environmental Monitoring Information Portal: http://donnees.ec.gc.ca/data/air/monitor/ambient-air-quality-oil-sands-region/pollutant-transformation-summer-2013-aircraft-intensive-multi-parameters-oil-sands-region/?lang = en The source data underlying Figs. 1–4, Supplementary Figs. 2b, c, 3d, 4b, and Tables 1 and 2 are provided as a Source Data file.

## Source data

Source Data

## Code availability

All the computer code associated with the TERRA algorithm, including for the kriging of pollutant data, a demonstration dataset and associated documentation is freely available upon request. The authors request that future publications which make use of the TERRA algorithm cite Gordon et al.^18^, Liggio et al.^34^, or Li et al.^17^ publications as appropriate.
