# Author Correction: Measured Canadian oil sands CO_2_ emissions are higher than estimates made using internationally recommended methods

**DOI:** 10.1038/s41467-022-35220-6

**Published:** 2022-11-29

**Authors:** John Liggio, Shao-Meng Li, Ralf M. Staebler, Katherine Hayden, Andrea Darlington, Richard L. Mittermeier, Jason O’Brien, Robert McLaren, Mengistu Wolde, Doug Worthy, Felix Vogel

**Affiliations:** 1 Air Quality Research Division, Environment and Climate Change Canada, 4905 Dufferin Street, Toronto, ON M3H 5T4 Canada; 2 Centre for Atmospheric Chemistry, York University, 4700 Keele Street, Toronto, ON M3J 1P3 Canada; 3 Flight Research Laboratory, National Research Council Canada, Ottawa, ON K1A 0R6 Canada; 4 Climate Research Division, Environment and Climate Change Canada, 4905 Dufferin Street, Toronto, ON M3H 5T4 Canada

Correction to: *Nature Communications* 10.1038/s41467-019-09714-9, published online 23 April 2019

The original version of this Article contained an error in Fig. 3d, in which the ratio of molecular weights of CO_2_ and SO_2_ were omitted in the calculations leading to 30% decrease in the CO_2_ upgrading stack emissions. The correct version of Fig. 3 is:


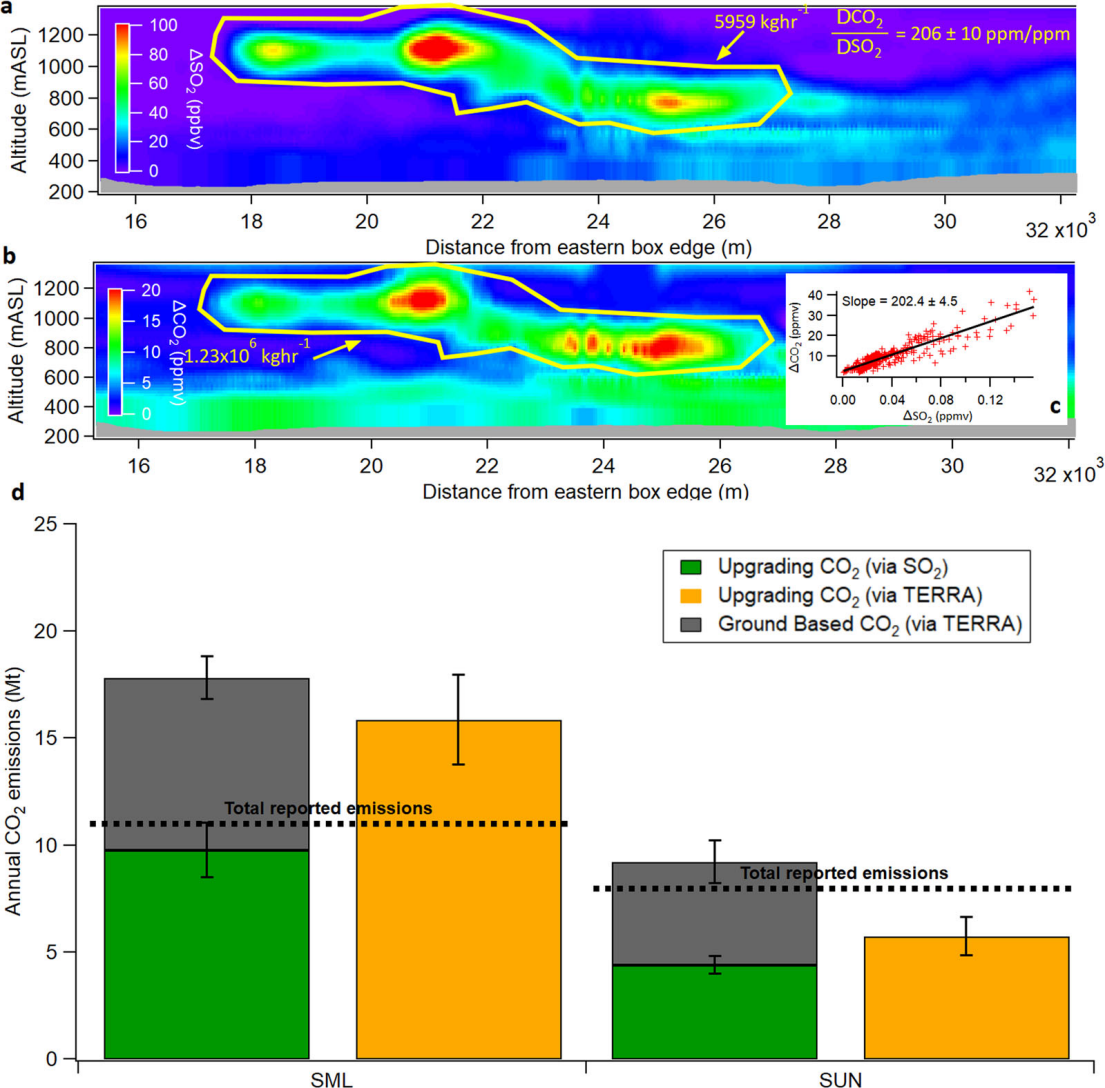


which replaces the previous incorrect version


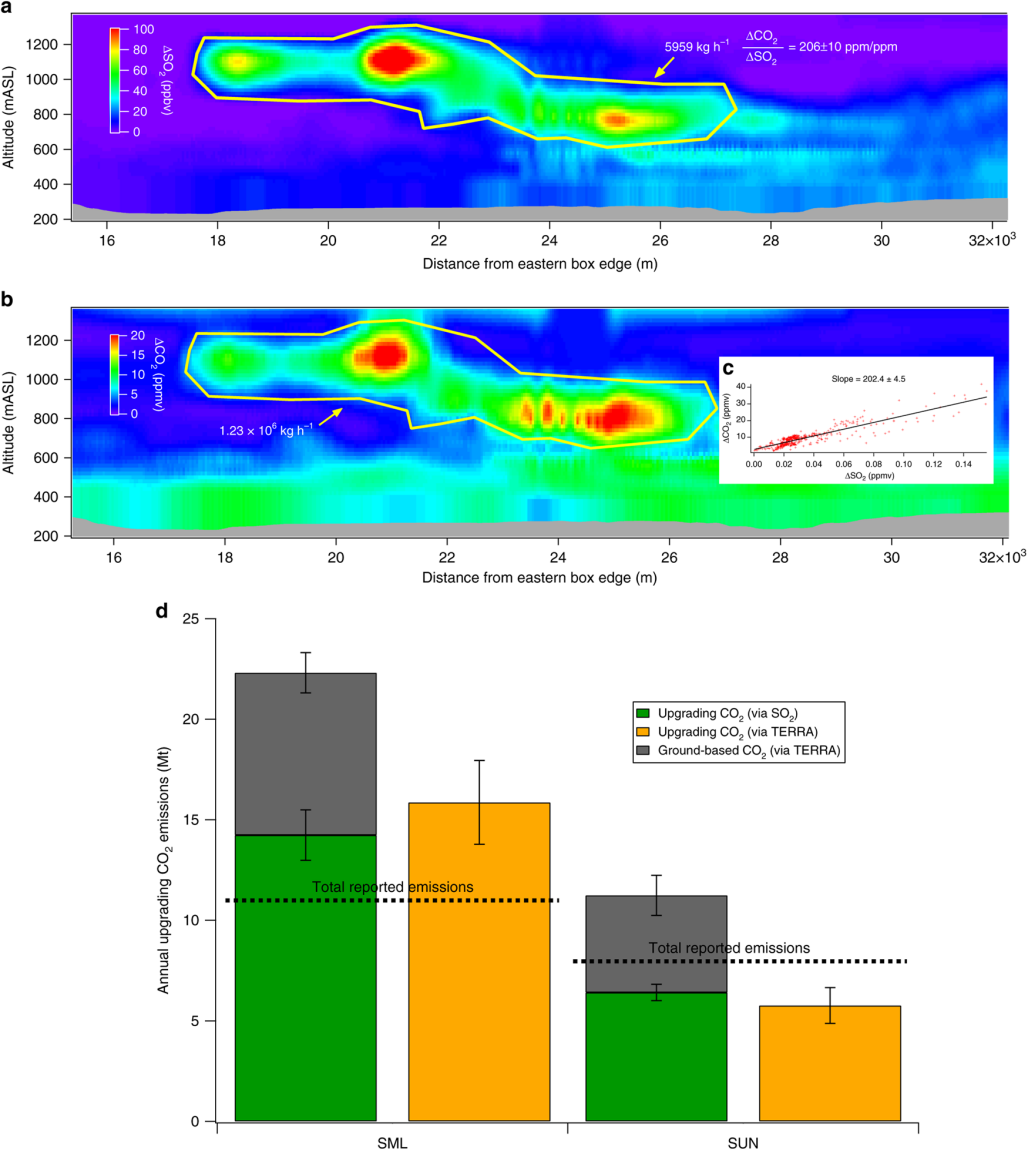


This has been corrected in both the PDF and HTML versions of the Article.

