# Supplementary material for: Measured Canadian oil sands CO2 emissions are higher than estimates made using internationally recommended methods
Source: Nat Commun. 2019 Apr 23;10:1863. doi: 10.1038/s41467-019-09714-9 (PMC6478833; doi:10.1038/s41467-019-09714-9)
Supplement: Supplementary file 1 — Supplementary Information [file 41467_2019_9714_MOESM1_ESM.pdf]

Supplementary Information

Measured Canadian Oil Sands CO_2 Emissions Are Higher than Estimates Made Using Internationally Recommended Methods

John Liggio et al.,

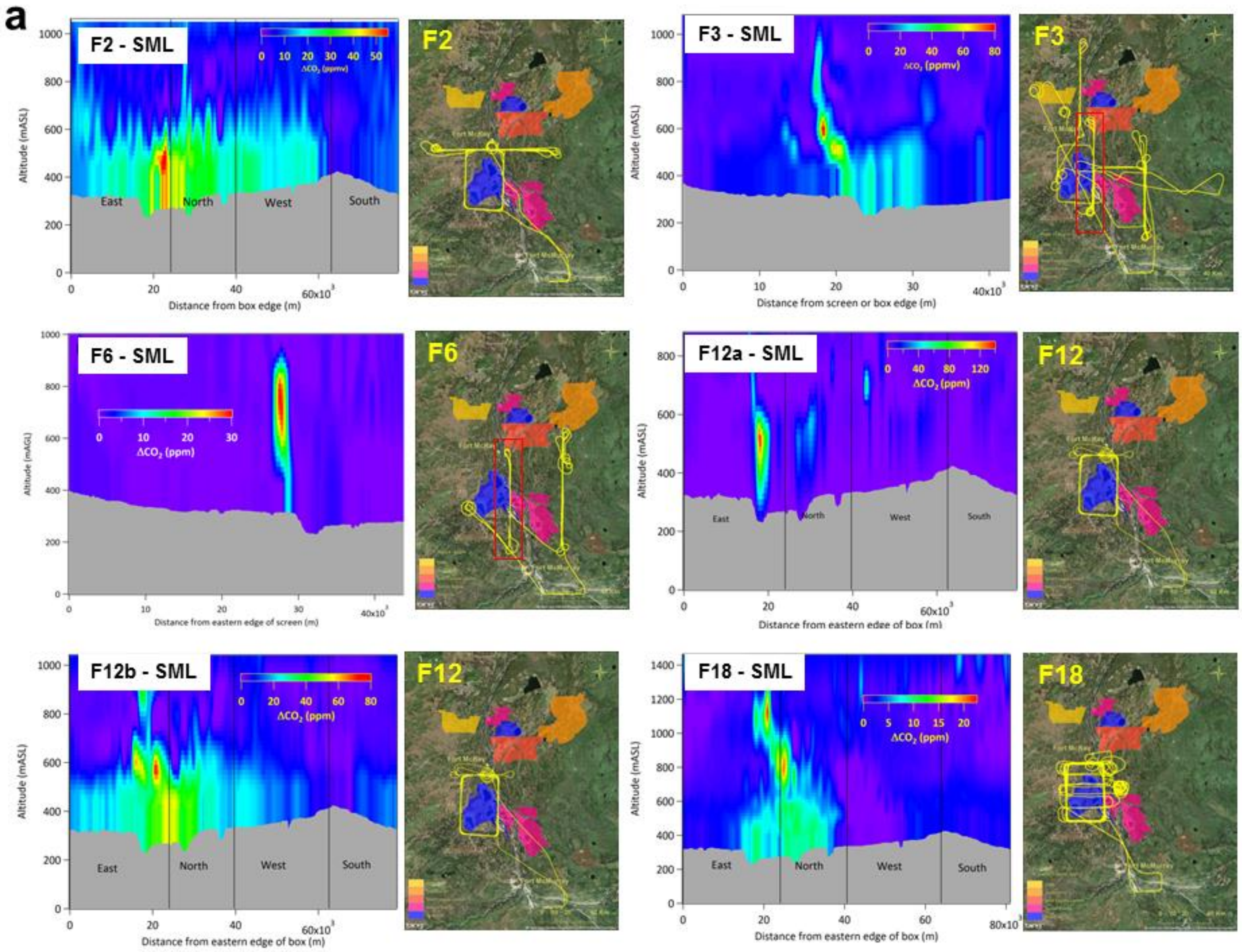
a
F2 - SML
F2
F3 - SML
F3
F6 - SML
F6
F12a - SML
F12
F12b - SML
F12
F18 - SML
F18
Altitude (mASL)
Distance from box edge (m)
Distance from screen or box edge (m)
Distance from eastern edge of screen (m)
Distance from eastern edge of box (m)
East
North
West
South
ΔCO2 (ppmv)
ΔCO2 (ppm)

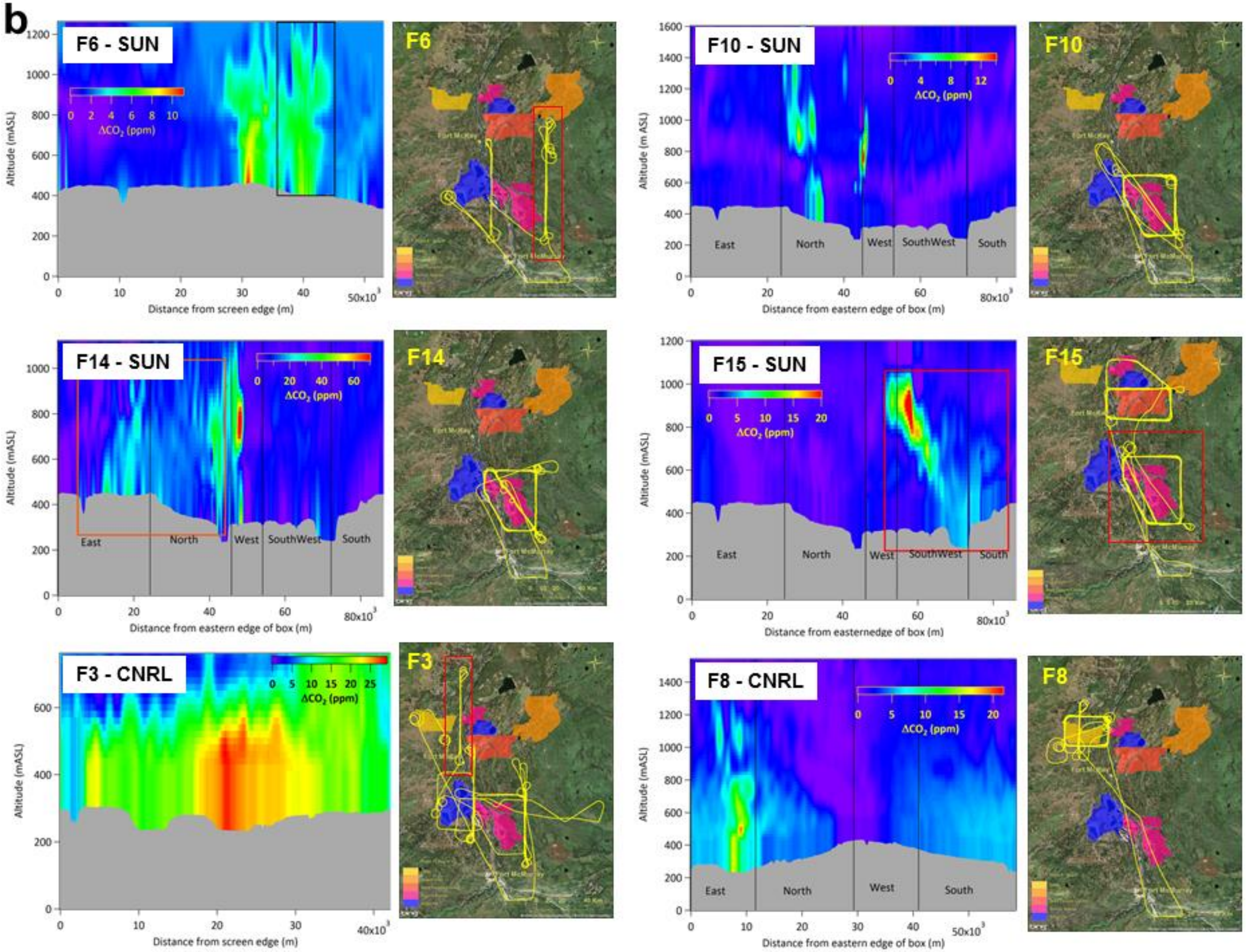
b
F6 - SUN
F6
F10 - SUN
F10
F14 - SUN
F14
F15 - SUN
F15
F3 - CNRL
F3
F8 - CNRL
F8
ΔCO2 (ppm)
Altitude (mASL)
Distance from screen edge (m)
Distance from eastern edge of box (m)
Distance from easternedge of box (m)
East
North
West
SouthWest
South

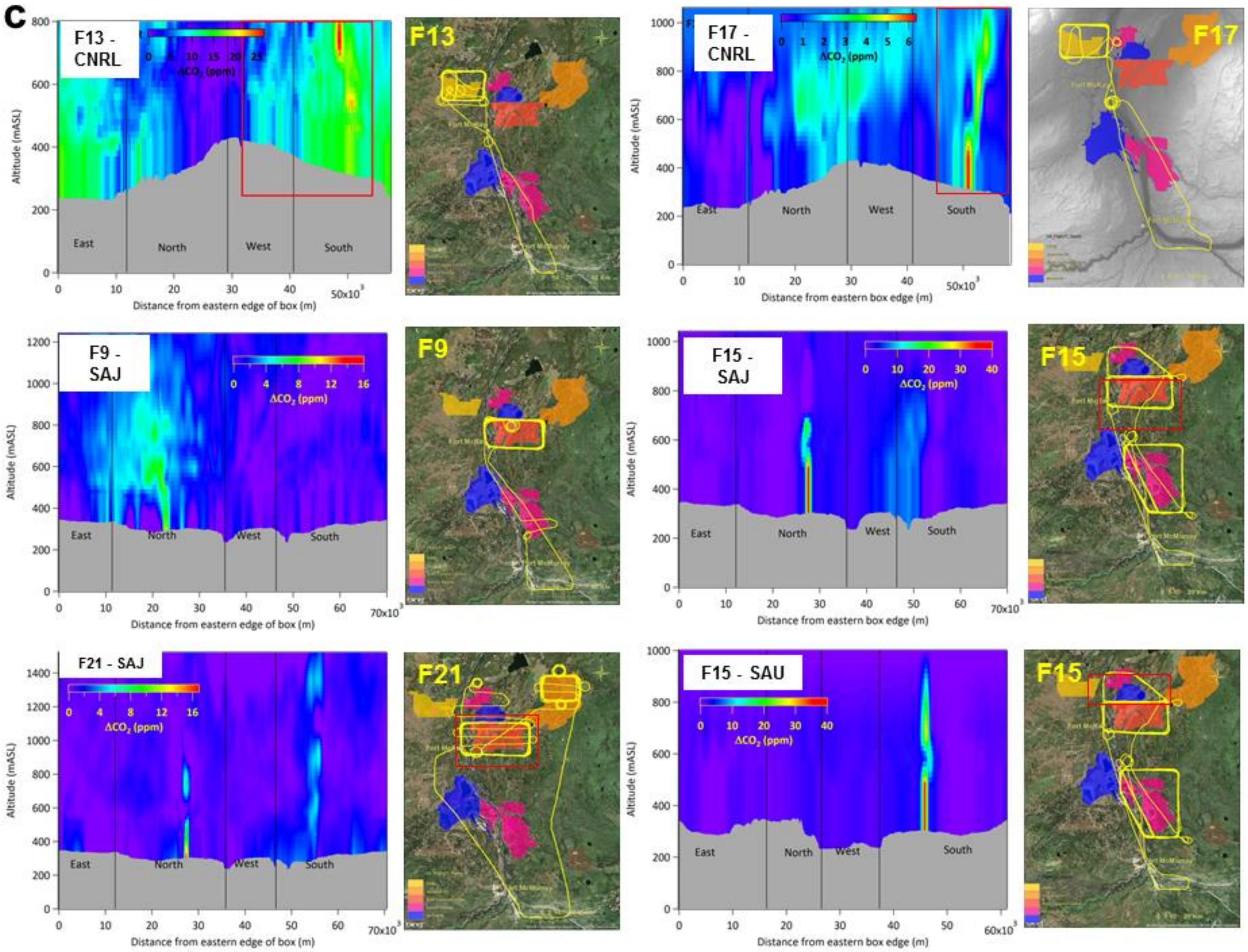

Supplementary Figure 1. Virtual CO_2 concentration polygons or screens derived by TERRA. Results shown in two dimensions, with associated flight paths for all of the flights used in the current work. Constant extrapolation used for concentrations below the lowest flight altitude. **a**. Flights for SML. **b** Flights for SUN and CNRL. **c**. Flights for CNRL, SAJ and SAU. Map data: Bing Maps, 2015.

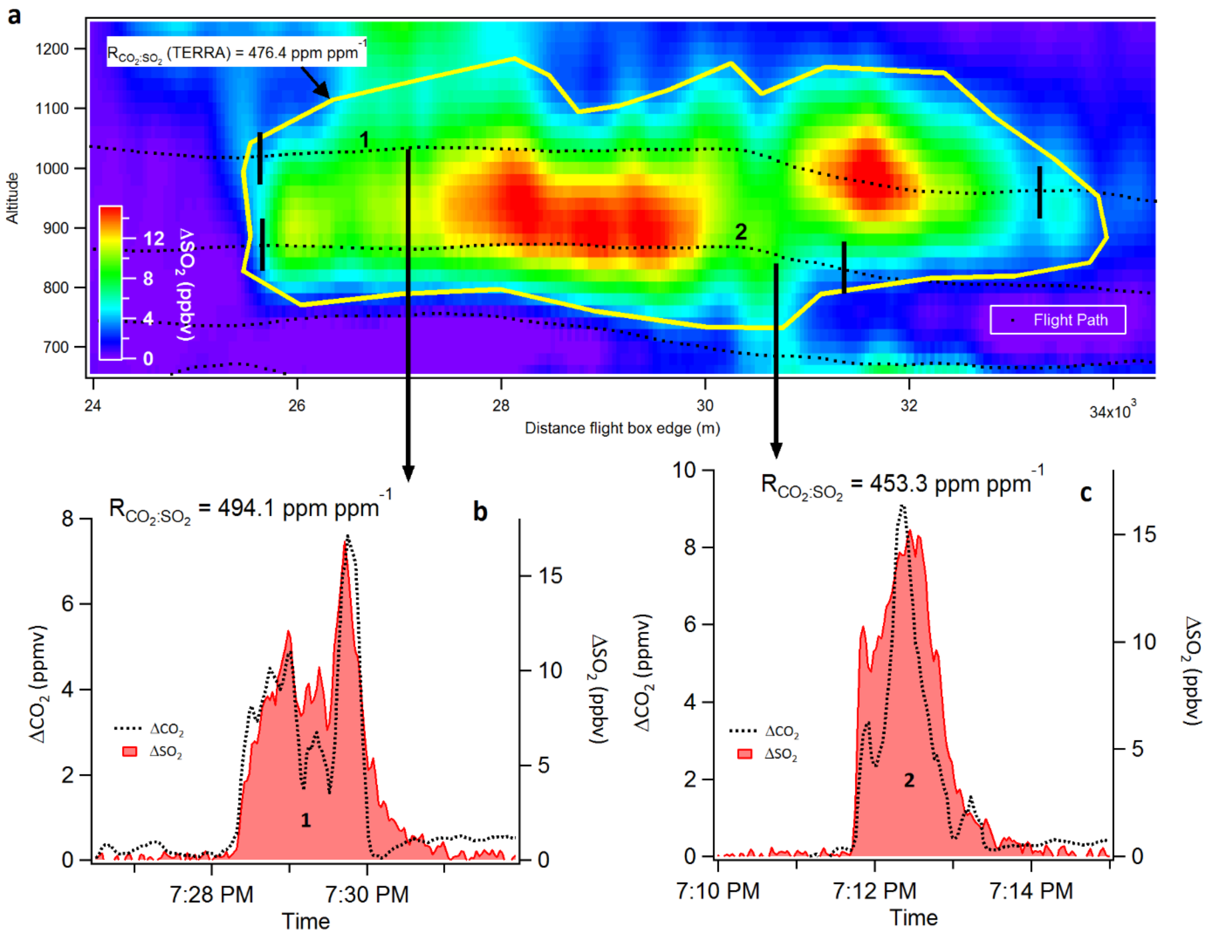

Supplementary Figure 2. CO_2 : SO_2 emission ratios for upgrading operations during flight 10 (SUN). **a**. Background subtracted SO_2 in elevated plumes from upgrading emissions during flight 10 derived with TERRA. Flight transects denoted as dashed black lines 1 and 2. **b**. Background subtracted CO_2 and SO_2 concentrations associated with transect 1 above. **c**. Background subtracted CO_2 and SO_2 concentrations associated with transect 2 above. Source data are provided in Supplementary Data 1.

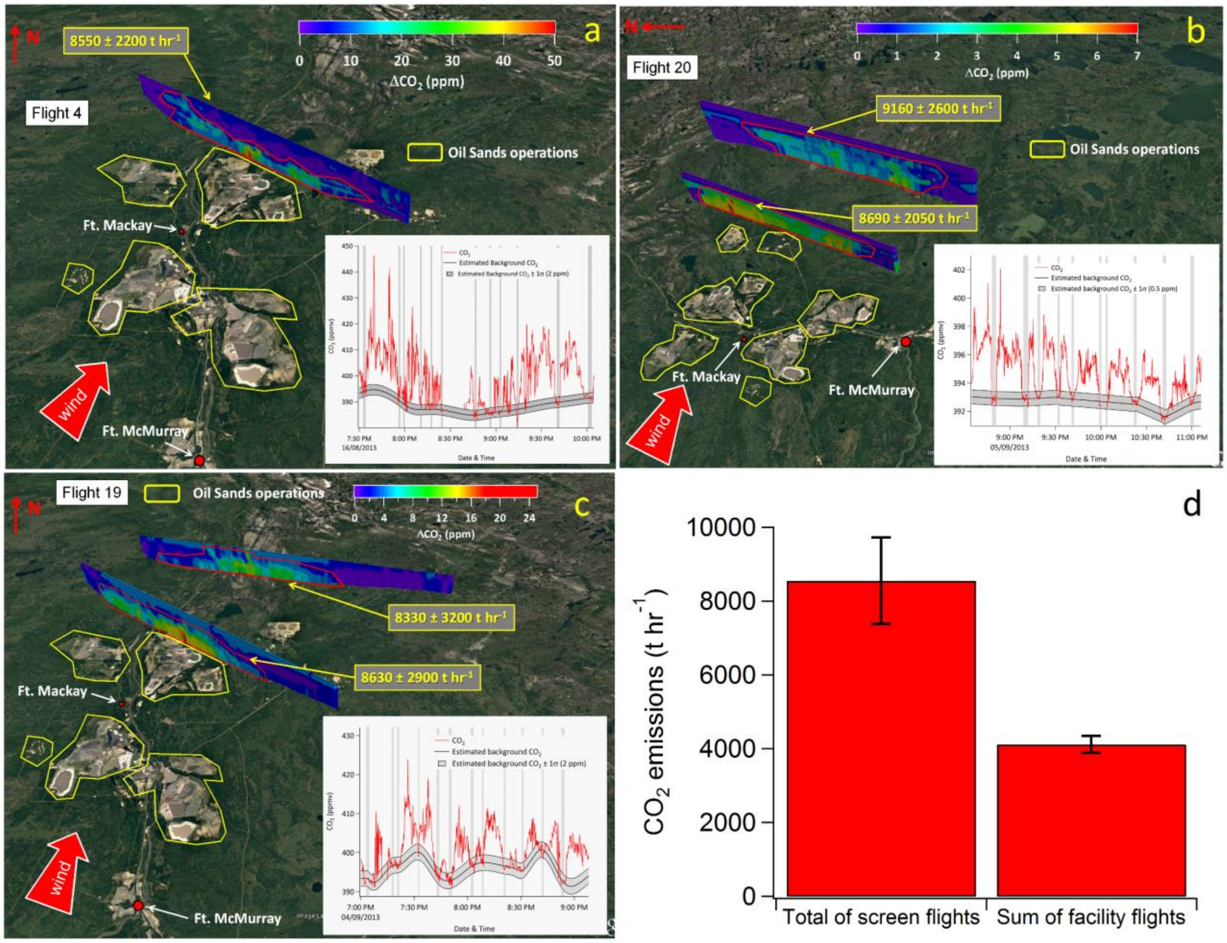

Supplementary Figure 3. Virtual CO_2 concentration screens for flights downwind of the OS. Background subtracted CO_2 concentration screen from **(a)** flight 4 **(b)**, flight 20 and **(c)** flight 19. Inset plots represent CO_2 time series used to derive the virtual screens, with estimated background CO_2 in grey. All times in UTC. **d**. Mean hourly CO_2 emission rate from screen flights 4, 19 and 20 compared to the mean hourly emission rate from the sum of individual facility flights. Error bars represent the propagated uncertainty in the mean. Source data for (**d**) are provided in Supplementary Data 1. Map data: Google, Image Landsat, CNES/Airbus, Digital Globe 2017

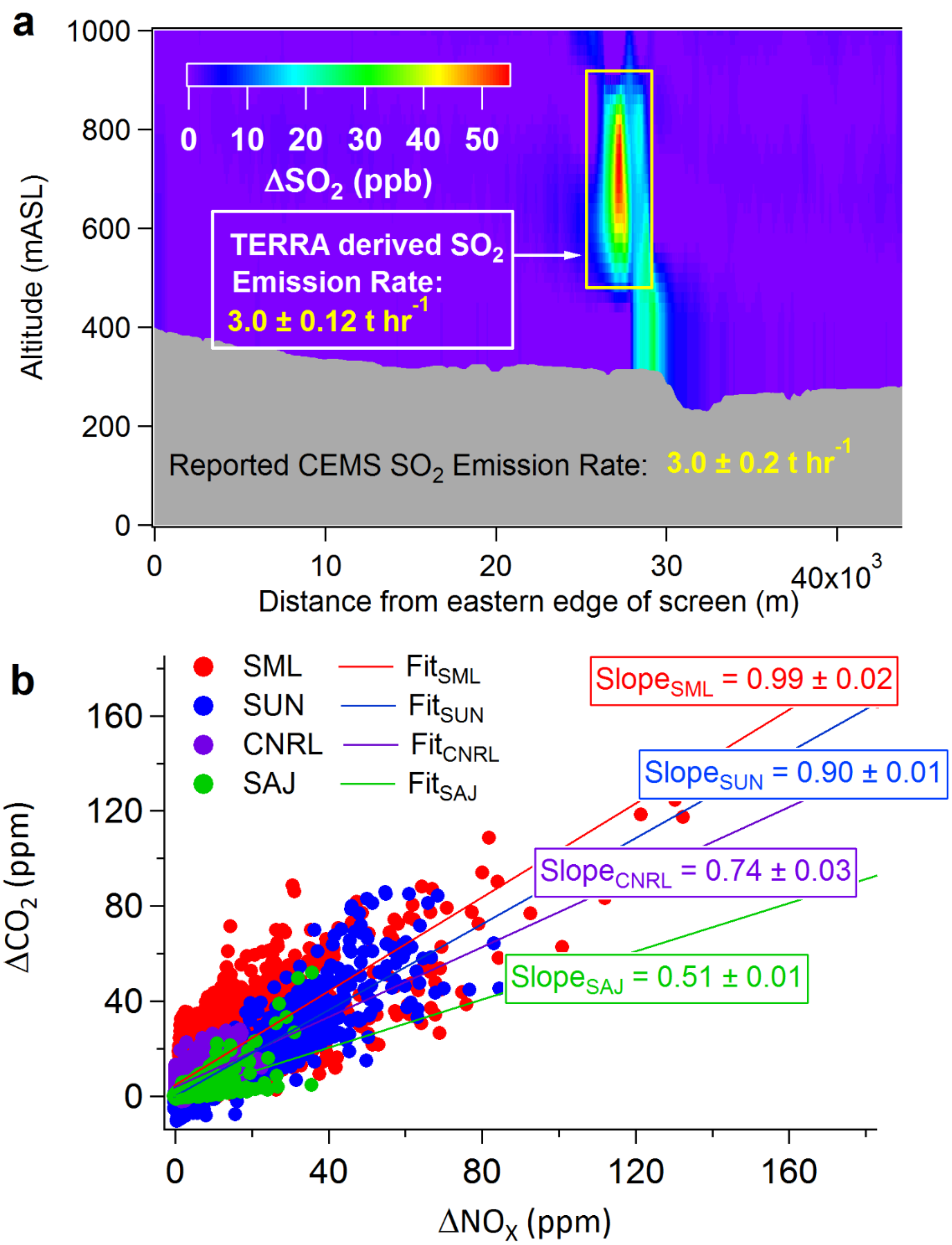

Supplementary Figure 4. Sulfur dioxide (SO_2) and Nitrogen oxides (NO_X) during Flight 6 over SML. **a**. Background subtracted SO_2 during flight 6 which exhibits a large plume from upgrading emissions. The hourly emission rate from TERRA compared to continuous emissions monitoring (CEMS) is also shown. **b**. Empirical relationship between background subtracted NO_X and CO_2 during all flights separated by facility. Error in slopes given as the standard deviation. Such relationships are the basis for an upscaling correction factor. Source data for (b) are provided in Supplementary Data 1.

Supplementary Table 1: Hourly CO_2 emissions derived with TERRA for individual facilities and transformation flights

Facility	Flight Num.	$\pm\delta_B$ (%)[a]	$\pm\delta_T$ (%)[b]	E_{CO_2} (t hr^{-1})[c]	$\pm\Delta E_{CO_2}$ (t hr^{-1})[d]	$\pm\Delta E_{CO_2}$ (%)
**Individual facility Flights**						
**SML**	2	17	20	1490	393	26
	3	18	7	1650	311	19
	6	17	7	1560	287	18
	12a	1	20	1800	359	20
	12b	13	20	1570	374	24
	18	8	8	1860	210	11
**Mean ± δ[e]**				**1650**	**134**	
**SUN**	6	19	20	1340	366	27
	10	14	8	1110	177	16
	14	18	20	1370	367	27
	15	2	7	1050	80	8
**Mean ± δ**				**1220**	**138**	
**CNRL**	3	20	20	487	138	28
	8	14	20	566	137	24
	13	11	20	514	118	23
	17	15	20	584	146	25
**Mean ± δ**				**538**	**68**	
**SAJ**	9	11	20	485	112	23
	15	9	20	445	98	22
	21	4	20	339	69	20
**Mean ± δ**				**423**	**55**	
**SAU**	15	4	20	285	58	21
**Transformation Flight Screens**						
**All surface mining**	4	16	20	8550	2223	26
**All surface mining**	19(a)	27	20	8630	2934	34
**All surface mining**	19(b)	33	20	8330	3165	38
**All surface mining**	20(a)	13	20	8690	2086	24
**All surface mining**	20(b)	20	20	9160	2565	28
**Mean ± δ**				**8672**	**1175**	
**Sum Facilities ± δ**	All			**4117**	**227**	

SML = Syncrude Mildred Lake facility, **SUN** =Suncor Millenium/Steepbank facility, **CNRL** = Horizon facility, **SAJ** = Shell Jack Pine and Muskeg River facilities, **SAU** = Syncrude Aurora facility**. a**. Error associated with background CO_2 determination (See methods). **b**. Error associated with the use of TERRA. **c**. Hourly CO_2 emissions derived with TERRA. **d.** Overall uncertainty in individual hourly CO_2 emissions calculated as $\Delta E_{CO_2} = \sqrt{\delta_B^2 + \delta_T^2}$

e. δ=propagated error of the mean $\delta = \frac{1}{N}\sqrt{\sum_{i=1}^{n} \Delta E_{CO_2 i}^2}$

Supplementary Table 2: Scaling factors, scaled monthly emissions, derived emissions intensities and annual CO_2 emissions for individual facility flights.

Facility	Flight Num.	$C_{monthly}$	$\pm\delta_C$	$E_{M_{CO_2}}$ (t month^{-1})	$\pm\Delta E_{M_{CO_2}}$ (t month^{-1})	I_{CO_2} (kg barrell^{-1})	$\pm\Delta I_{CO_2}$ (kg barrell^{-1})	E_{Annual} (t yr^{-1})	$\pm\delta_E$ (t yr^{-1})
**Individual Facility flights**									
SML[a]	2	0.702	0.100	$1.58x10^6$	$4.73x10^5$	250.9	76.0	$2.5x10^7$	$7.6x10^6$
	3	0.717	0.102	$1.71x10^6$	$4.03x10^5$	269.2	64.3	$2.7x10^7$	$6.5x10^6$
	6	1.136	0.162	$1.02x10^6$	$2.40x10^5$	174.0	41.4	$1.7x10^7$	$4.2x10^6$
	12a	0.725	0.103	$1.84x10^6$	$4.53x10^5$	287.8	71.7	$2.9x10^7$	$7.2x10^6$
	12b	0.725	0.103	$1.61x10^6$	$4.47x10^5$	255.2	71.7	$2.6x10^7$	$7.2x10^6$
	18	0.956	0.137	$1.45x10^6$	$2.65x10^5$	232.8	43.7	$2.3x10^7$	$4.4x10^6$
**Mean±δ[b]**		**0.83**	**0.05**	**$1.54x10^6$**	**$1.60x10^5$**	**245.0**	**25.7**	**$2.5x10^7$**	**$2.6x10^6$**
SUN	6	1.025	0.145	$9.73x10^5$	$2.99x10^5$	92.5	29.2	$9.8x10^6$	$3.1x10^6$
	10	1.027	0.146	$8.05x10^5$	$1.73x10^5$	76.6	17.3	$8.1x10^6$	$1.8x10^6$
	14	1.014	0.144	$1.01x10^6$	$3.05x10^5$	96.0	29.8	$1.0x10^7$	$3.1x10^6$
	15	0.963	0.137	$8.10x10^5$	$1.28x10^5$	77.1	13.4	$8.1x10^6$	$1.4x10^6$
**Mean±δ**		**1.00**	**0.07**	**$8.99x10^5$**	**$1.20x10^5$**	**85.5**	**11.8**	**$9.0x10^6$**	**$1.2x10^6$**
CNRL	3	1.024	0.149	$3.54x10^5$	$1.12x10^5$	103.6	33.1	$3.8x10^6$	$1.2x10^6$
	8	0.726	0.106	$5.80x10^5$	$1.63x10^5$	170.0	48.2	$6.3x10^6$	$1.8x10^6$
	13	0.990	0.144	$3.86x10^5$	$1.04x10^5$	113.2	30.9	$4.2x10^6$	$1.1x10^6$
	17	0.961	0.136	$4.52x10^5$	$1.30x10^5$	126.7	36.8	$4.7x10^6$	$1.4x10^6$
**Mean±δ**		**0.925**	**0.06**	**$4.43x10^5$**	**$6.46x10^4$**	**128.4**	**18.9**	**$4.7x10^6$**	**$6.9x10^5$**
SAJ	9	0.906	0.130	$3.98x10^5$	$1.08x10^5$	50.8	14.7	$4.4x10^6$	$1.3x10^6$
	15	0.906	0.130	$3.66x10^5$	$9.54x10^4$	46.6	13.1	$4.0x10^6$	$1.1x10^6$
	21	0.906	0.130	$2.78x10^5$	$6.90x10^4$	35.5	9.5	$3.1x10^6$	$8.3x10^5$
**Mean±δ**		**0.906**	**0.130**	**$3.47x10^5$**	**$5.31x10^4$**	**44.3**	**7.2**	**$3.9x10^6$**	**$6.3x10^5$**
SAU[c]	15	0.906	0.130	$2.34x10^5$	$5.65x10^4$	-	-	-	-

Description of the derivation of Monthly scaling factors**,** monthly emission rates of CO_2, CO_2 emission intensities, annual CO_2 emissions and associated uncertainties is provided in methods. **a.** SML includes monthly CO_2 emissions from SAU. **b.** δ= propagated error of the mean, eg: $\delta = \frac{1}{N}\sqrt{\sum_{i=1}^{n} \Delta E_{\mathrm{m}_{\mathrm{CO_2}}\mathrm{i}}^2}$

c. SAU emission intensity and annual emissions are not calculated as it does not produce SCO and since its emissions are included in SML. $C_{monthly}$ for SAU is taken to be the mean of the other facilities as no CEMS data is available, with uncertainty equal to that of SAJ.

Supplementary Note 1

Contribution of local highway vehicle emissions

Emission flights for SML and SUN facilities resulted in virtual boxes containing short sections of the main highway of the region (HWY 63) as described elsewhere[1]. In principle, CO_2 emissions from the vehicles on the road could contribute to the TERRA derived emissions during the flights. Since much of the CO_2 from these 2 facilities arise from the elevated upgrader stacks (61- 67%), ground based vehicular CO_2 is not likely to influence overall facility emissions. Regardless, the influence of on-road CO_2 on the hourly derived total emission rates is estimated using measured traffic flow for the section of highway within the virtual box[2] and the average fuel efficiency of the vehicles[3]. The hourly CO_2 emissions from vehicles on the road ($V_{\mathrm{CO_2}}$) and within the virtual boxes can be given by

$$V_{CO_2} = \frac{V_{\mathrm{fe}} * L * \rho_{\mathrm{fuel}} * f_{\mathrm{C}} * \frac{MW_{\mathrm{CO_2}}}{MW_{\mathrm{C}}} * T_{\mathrm{flow}}}{1000} \tag{11}$$

Where V_{fe} represents the average fuel efficiency of the vehicles (L per 100 km), L is the length of road present within the virtual box (km), ρ_{fuel} is the fuel density (≈0.8 kg L^{-1}), f_{c} is the carbon mass fraction of the fuel (≈0.8), MW is the molecular weight of C and CO_2, T_{flow} is the traffic flow (vehicles hr^{-1}) and the factor of 1000 converts kg to tonnes.

During SUN flights, 13 km of HWY 63 (L) was located within the virtual boxes and approximately 600 vehicles hr^{-1} (T_{flow}) travelled through this distance during the flight hours (≈11:00 - 13:00 MDT), of which ≈75% were light duty gasoline powered and ≈25% heavy duty diesel[2]. Assuming a weighted average fuel efficiency of 15.5 L per 100km, and complete combustion to CO_2, approximately 2.8 tonnes hr^{-1} of CO_2 could have been emitted into the virtual boxes for SUN. Similarly for SML flights, 17 km of the HWY was present within the virtual boxes, and approximately 300 vehicles hr^{-1} passed through this distance, resulting in approximately 1.9 tonnes hr^{-1} of CO_2 emitted from on-road vehicles. These estimated vehicular CO_2 emissions are very small and not resolvable with TERRA. Given the total hourly CO_2 emissions derived by TERRA for SUN and SML, the estimated vehicular emissions of CO_2 are negligible, contributing less than 0.25% and 0.11% respectively to the hourly emissions of Figure 1.

Supplementary Note 2.

CO_2 emissions through the use of emission ratios

The large majority of SO_2 emissions from oil sands surface mining activities are associated with the upgrading of raw bitumen material. Such SO_2 emissions are generally confined to relatively few elevated stacks which are measured via continuous emissions monitoring systems (CEMS), audited, and used as the basis of SO_2 emission reporting to the National Pollution Release Inventory (NPRI)[4]. These stacks range in height from ≈70 - 183 m above ground resulting in measureable elevated plumes of SO_2 which are clearly distinguishable from ground based

sources. The emissions of SO_2 from these stacks is accompanied by CO_2, providing an opportunity to quantify the emissions of CO_2 from upgrading processes. In principle, the annual emissions of CO_2 from the elevated stacks $\left(E_{\mathrm{STACK,CO_2}}\right)$ can be derived from the ratio of CO_2 to SO_2 in the plumes via

$$E_{\mathrm{STACK,CO_2}} = Rt_{\mathrm{CO_2:SO_2}} \times E_{\mathrm{SO_2}} \tag{12}$$

where $Rt_{\mathrm{CO_2:SO_2}}$ is the ratio of CO_2 to SO_2 within the elevated stack plumes and $E_{\mathrm{SO_2}}$ is the annual emissions of SO_2 reported to the NPRI[4] for a given facility (63132 t for SML and 14104 t for SUN). $R_{\mathrm{CO_2:SO_2}}$ was determined for the SML and SUN facilities using flights where the stack plumes were clearly observable, by utilizing both the TERRA derived CO_2 and SO_2 plume emissions, and the direct concentration measurement ratios (flights 3, 10, 12, 18 for SML and flights 10, 15 for SUN). Using the TERRA algorithm, the transfer rates for SO_2 and CO_2 in the same elevated plumes were quantified by integrating subsections of the TERRA virtual screens (Figure 3a,b and Supplementary Figure 2a) to derive $R_{\mathrm{CO_2:SO_2}}$. Alternatively, the time integrated measured concentrations of CO_2 and SO_2 along the flight tracks through these plumes were also used to derive $R_{\mathrm{CO_2:SO_2}}$ as shown in Supplementary Figure 2b,c. Both approaches are consistent with each other, yielding values of $R_{\mathrm{CO_2:SO_2}}$ within <5% of each other, with little variation between different flights on separate days. The final values of $R_{\mathrm{CO_2:SO_2}}$ used in determining annual emissions were taken as the mean of the ratios across the various flights for a given facility, using both approaches. Accordingly, the derived values of $R_{\mathrm{CO_2:SO_2}}$ were 225.6±20 ppm CO_2 ppm SO_2^{-1} and 466.3±28.2 ppm CO_2 ppm SO_2^{-1} for SML and SUN respectively.

Emissions from ground based sources were quantified similarly, using the TERRA derived hourly emissions for portions of the virtual box that were clearly not impacted by elevated stack plumes (ie: F3, F6, F18 for SML and F10 and F15 for SUN). These hourly emissions were scaled up to one month as described above, and normalized by reported OS ore mined for the same month[5]. The annual CO_2 emissions from these ground sources $(E_{\mathrm{ground,CO_2}})$ are then derived as

$$E_{\mathrm{ground,CO_2}} = Rt_{\mathrm{CO_2:Ore}} \times \text{mined ore} \tag{13}$$

where $Rt_{\mathrm{CO_2:Ore}}$ is the ratio of TERRA derived (scaled to the month) to reported mined OS ore. The ground based CO_2 emission ratios derived for SML and SUN were 88.6±10.6 and 32.0±4.5 kg m^{-3} mined ore respectively.

Supplementary Note 3.

Transformation flights and TERRA

As noted above, three flights (F4, F19, and F20) were conducted to quantify emissions from the OS in its entirety (for comparison to the sum of individual emission flights), as they encompass the majority of emissions from the surface mining region. The virtual screens derived with TERRA from these flights are shown in Supplementary Figure 3a-c. The CO_2 transfer rates

across these screens ranged from 8300±3200 t hr^{-1} to 9200±2600 t hr^{-1} and were highly consistent between flights, between screens within a flight, and across several days spanning a month. The uncertainties associated with such screens are higher than those of individual facility flights (26-38%; Supplementary Table 1), due mainly to the more variable background CO_2 expected as a result of increased flight time length, and by being limited to using the edge of screens rather than upwind transects. Nonetheless, the mean hourly transfer rate from these flights was 8676 t hr^{-1} ±1200 t hr^{-1} where the uncertainty (δ) is given as $= \frac{1}{N}\sqrt{\sum_{i=1}^{n} \Delta E_{CO_2 i}^2}$. The mean hourly transfer rate for these flights was significantly higher than the hourly emission rates derived as the sum of individual facility flights (Supplementary Figure 3d and Supplementary Figure 1). This discrepancy suggests that there are additional CO_2 sources, such as the in-situ and cogeneration facilities of Suncor-MacKay River as well as those of Husky Sunrise and Suncor-Firebag which would be captured in these flight screens. CO_2 emissions from the small town of Fort Mckay are also likely included, although such emissions are expected to be small from this community of ~560 people. Emissions from the larger town of Fort McMurray to the south are not likely to influence the derived hourly rates, since the town was not upwind of the flight screens, and the observed rates were not significantly different between F7, F19 and F20 despite differences in wind direction (Supplementary Figure 3a-c). Biogenic emissions of CO_2 may also be present between these screens, although vegetation is more likely to take up CO_2 rather than release it during the day. As a result, the most likely explanation for these elevated CO_2 hourly emissions is the inclusion of additional anthropogenic OS related sources. Given the magnitude of the hourly CO_2 emissions in downwind transects (a factor of two higher than the sum of surface mining/upgrading emissions; Supplementary Figure 3d), the few in-situ/cogeneration facilities within the measurement domain noted above are not likely sufficient to account for this difference. This is however, consistent with elevated BC emissions rates downwind of the OS, from the same screens and may be indicative of combustion processes which are not included in the individual facility flights (ie: outside of their boundaries) including the burning of overburden. These results suggest that annual emissions of CO_2 from the OS may be even greater than those shown in Fig 4, as the transformation flight screens did not include the majority of in-situ facilities south of Fort McMurray, and because hourly emission rates were highly consistent downwind for flights spanning one month.

Supplementary References

1 Li, S.-M. *et al.* Differences between Measured and Reported Volatile Organic Compound Emissions from Oil Sands Facilities in Alberta, Canada *Proc. Natl. Acad. Sci. U.S.A.* **114**, E3756-E3765, doi: 10.1073/pnas.1617862114 (2017).

2 Government of Alberta. Traffic Counts. (2013). <http://www.transportation.alberta.ca/3459.htm>.

3 Statistics Canada. Canadian Vehicle Survey 2009. (2010). <http://www5.statcan.gc.ca/olc-cel/olc.action?objId=53-223-X&objType=2&lang=en&limit=1>

4 Environment and Climate Change Canada. National Pollution Release Inventory. (2013). <https://www.canada.ca/en/services/environment/pollution-waste-management/national-pollutant-release-inventory.html>

5 Alberta Energy Regulator, Alberta Mineable Oil Sands Plant Statistics. (2013). <https://www.aer.ca/data-and-publications/statistical-reports/st39>
