# Supplementary material for: Measured Canadian oil sands CO2 emissions are higher than estimates made using internationally recommended methods
Source: Nat Commun. 2019 Apr 23;10:1863. doi: 10.1038/s41467-019-09714-9 (PMC6478833; doi:10.1038/s41467-019-09714-9)
Supplement: Supplementary file 2 — Reporting Summary [file 41467_2019_9714_MOESM2_ESM.pdf]

nature research

Corresponding author(s): John Liggio and Shao-Meng Li

Last updated by author(s): Feb 28, 2019

Reporting Summary

Nature Research wishes to improve the reproducibility of the work that we publish. This form provides structure for consistency and transparency in reporting. For further information on Nature Research policies, see Authors & Referees and the Editorial Policy Checklist.

Statistics

For all statistical analyses, confirm that the following items are present in the figure legend, table legend, main text, or Methods section.

n/a	Confirmed	
☐	☒	The exact sample size (*n*) for each experimental group/condition, given as a discrete number and unit of measurement
☒	☐	A statement on whether measurements were taken from distinct samples or whether the same sample was measured repeatedly
☐	☒	The statistical test(s) used AND whether they are one- or two-sided<br>*Only common tests should be described solely by name; describe more complex techniques in the Methods section.*
☒	☐	A description of all covariates tested
☐	☒	A description of any assumptions or corrections, such as tests of normality and adjustment for multiple comparisons
☐	☒	A full description of the statistical parameters including central tendency (e.g. means) or other basic estimates (e.g. regression coefficient) AND variation (e.g. standard deviation) or associated estimates of uncertainty (e.g. confidence intervals)
☐	☒	For null hypothesis testing, the test statistic (e.g. F, t, r) with confidence intervals, effect sizes, degrees of freedom and P value noted<br>*Give P values as exact values whenever suitable.*
☒	☐	For Bayesian analysis, information on the choice of priors and Markov chain Monte Carlo settings
☒	☐	For hierarchical and complex designs, identification of the appropriate level for tests and full reporting of outcomes
☒	☐	Estimates of effect sizes (e.g. Cohen's *d*, Pearson's *r*), indicating how they were calculated

Our web collection on statistics for biologists contains articles on many of the points above.

Software and code

Policy information about availability of computer code

Data collection	All data was collected with several different commercially available atmospheric pollutant measuring instruments, each with their own proprietary source code and software.
Data analysis	Data analysis was performed with IGOR Pro Version 6.0 (Wavemetrics Inc), with code for emissions estimation from aircraft data written in IGOR Pro 6.0. The source code for the TERRA algorithm written with IGOR Pro is provided in a Supplementary File.

For manuscripts utilizing custom algorithms or software that are central to the research but not yet described in published literature, software must be made available to editors/reviewers. We strongly encourage code deposition in a community repository (e.g. GitHub). See the Nature Research guidelines for submitting code & software for further information.

Data

Policy information about availability of data

All manuscripts must include a data availability statement. This statement should provide the following information, where applicable:

- Accession codes, unique identifiers, or web links for publicly available datasets
- A list of figures that have associated raw data
- A description of any restrictions on data availability

All data used in this publication are freely available on the Canada-Alberta Oil Sands Environmental Monitoring Information Portal: http://donnees.ec.gc.ca/data/air/monitor/ambient-air-quality-oil-sands-region/pollutant-transformation-summer-2013-aircraft-intensive-multi-parameters-oil-sands-region/?lang=en The source data underlying Figs 1-4, Supplementary figures 2d, 3b, 4b, 4c and Tables 1 and 2 are provided as a Source Data file.

Field-specific reporting

Please select the one below that is the best fit for your research. If you are not sure, read the appropriate sections before making your selection.

☐ Life sciences ☐ Behavioural & social sciences ☒ Ecological, evolutionary & environmental sciences

For a reference copy of the document with all sections, see nature.com/documents/nr-reporting-summary-flat.pdf

Ecological, evolutionary & environmental sciences study design

All studies must disclose on these points even when the disclosure is negative.

Study description	Measurements of Carbon dioxide (CO2) in the oil Sands region of Canada, using an aircraft platform. Data are used to derive emissions for specific oil sands facilities and the overall emissions for the oil sands in general.
Research sample	The sample in this case is simply the air surrounding the oil sands, which contains CO2.
Sampling strategy	Sample size (ie: number of flights and length of flights) was determined based primarily on the amount of fuel in the aircraft required to fly a full box around a given industrial facility, and the number of flight hours allooted for the project overall.
Data collection	Measurements of CO2 were made aboard the aircraft with a commercial Piccarro CRD instrument, with 2 second time resolution for the duration of a given flight.
Timing and spatial scale	Measurements were made on various days during the months of august and September, 2013. Each flight was conducted for approximately 2-5 hours depending on the length of time required to completely encircle a given facility. Each box shaped flight covered an area of approximately 20km x 20km depending upon the size of the given oil sands facility.
Data exclusions	Data were excluded when the measured wind direction or wind speed during flight were not conducive to the estimation of total emissions from a facility, or when emissions from neighboring facilities unduly influenced the emissions data from the facility being studied. These cases were assessed after the study and during analysis of data.
Reproducibility	Between 3 and 6 flights around each facility were conducted, to optimally make use of the prescribed number of flight hours.
Randomization	Flights around each facility were generally conducted when the meteorology was suitable for the experiment, and were not randomized intentionally.
Blinding	This is not relevant for this study as it is an aircraft campaign where all industries being investigated are known.

Did the study involve field work? ☒ Yes ☐ No

Field work, collection and transport

Field conditions	Aircraft data was collected on rain free days, with favorable meteorology, and minimal clouds.
Location	Oil sands region north of Fort McMurray, Alberta, Canada, covering an area of approximately 60 km x 80 km.
Access and import/export	not relevant for this study
Disturbance	not relevant for this study

Reporting for specific materials, systems and methods

We require information from authors about some types of materials, experimental systems and methods used in many studies. Here, indicate whether each material, system or method listed is relevant to your study. If you are not sure if a list item applies to your research, read the appropriate section before selecting a response.

Materials & experimental systems

n/a	Involved in the study
☒	☐ Antibodies
☒	☐ Eukaryotic cell lines
☒	☐ Palaeontology
☒	☐ Animals and other organisms
☒	☐ Human research participants
☒	☐ Clinical data

Methods

n/a	Involved in the study
☒	☐ ChIP-seq
☒	☐ Flow cytometry
☒	☐ MRI-based neuroimaging
